# Early Identification and Prognostic Stratification of Cancer Cachexia Using Explainable Machine Learning: A Multicentre Cohort Study

**DOI:** 10.1002/jcsm.70370

**Published:** 2026-08-21

**Authors:** Wei Huang, Kan Pan, Mingjian Zhao, Tingting Zhao, Nuo Xu, Hanping Shi

**Affiliations:** ^1^ Institute of Clinical Nutrition and Department of Colorectal Surgery The First Affiliated Hospital of Wenzhou Medical University Wenzhou Zhejiang China; ^2^ The First School of Medicine and School of Information and Engineering Wenzhou Medical University Wenzhou Zhejiang China; ^3^ Zhejiang Key Laboratory of Intelligent Cancer Biomarker Discovery and Translation The First Affiliated Hospital, Wenzhou Medical University Wenzhou China; ^4^ Department of Gastrointestinal Surgery/Department of Clinical Nutrition, Beijing Shijitan Hospital Capital Medical University Beijing China; ^5^ Key Laboratory of Cancer Foods for Special Medical Purpose (FSMP) for State Market Regulation Beijing China; ^6^ Department of Gastrointestinal and Gland Surgery The First Affiliated Hospital, Guangxi Medical University Nanning Guangxi China

**Keywords:** cancer cachexia, explainable artificial intelligence, machine learning, prognostic stratification, real‐world data, survival prediction

## Abstract

**Background:**

Cancer cachexia is a heterogeneous syndrome that remains frequently underrecognized in routine oncology care, especially before overt wasting develops. We aimed to develop and externally validate an explainable two‐stage machine learning framework for early cachexia identification and prognostic stratification using routinely available clinical data.

**Methods:**

In this multicentre real‐world study based on the INSCOC registry, 10 796 hospitalized adults with cancer were analysed, including 3995 patients with cachexia and 6801 without cachexia. First‐stage machine learning models were trained separately in high‐ and low‐prevalence settings to identify cachexia. Second‐stage survival models were developed in 1711 cachexia patients with follow‐up data from a 5172‐patient follow‐up cohort. A total of 586 deaths were observed in the cachexia follow‐up cohort. External validation used an independent Fujian cohort of 886 patients.

**Results:**

Cachexia prevalence was 37.0% (3995/10 796). Compared with non‐cachexia patients, cachexia patients had lower median BMI (21.0 vs. 23.4 kg/m2), albumin (37.9 vs. 39.7 g/L), triceps skinfold thickness (13.0 vs. 16.0 mm), calf circumference (33.0 vs. 35.0 cm) and higher CRP (5.9 vs. 3.9 mg/L); all *p* < 0.001. For cachexia identification, XGBoost achieved the best discrimination, with an AUC of 0.867 (95% CI: 0.857–0.878), sensitivity 0.706 and specificity 0.846 in the high‐prevalence group, and an AUC of 0.870 (95% CI: 0.861–0.880), sensitivity 0.817 and specificity 0.756 in the low‐prevalence group. In external validation, the AUCs were 0.744 in the high‐prevalence subgroup and 0.694 in the low‐prevalence subgroup. Among cachexia patients with follow‐up, the 1‐year mortality rate was 20.8% (95% CI: 19.0%–22.9%). For prognostic stratification, the random survival forest model showed the most favourable overall performance, with a C‐index of 0.680 and time‐dependent AUCs of 0.800, 0.777, 0.741 and 0.716 at 3, 6, 9 and 12 months, respectively. Advanced TNM stage and higher CRP were associated with worse survival, whereas higher albumin, prealbumin and HDL were associated with better survival.

**Conclusions:**

This explainable two‐stage framework enables accurate cachexia identification across different prevalence settings and clinically meaningful prognostic stratification in patients with established cachexia. Using routine variables and independent external validation, it provides a practical basis for earlier recognition, risk‐adapted supportive care and precision management in oncology practice.

## 1 Introduction

Cancer cachexia is a multifactorial syndrome characterized by involuntary weight loss and progressive depletion of skeletal muscle and adipose tissue [1]. It is prevalent across various cancer types and stages, with an estimated prevalence ranging from 35% to 80% and is particularly common in patients with advanced or terminal disease [2]. This condition severely compromises patients' nutritional status, quality of life and tolerance of antitumor therapies while elevating the risk of complications, mortality and healthcare costs [3]. Approximately 20%–30% of cancer‐related deaths are attributed to cachexia [4]. However, due to limitations in current diagnostic methods, its actual clinical impact is often underestimated. Therefore, achieving early identification and proactive intervention is of great significance for improving prognosis.

In recent years, machine learning (ML) has shown significant potential in oncology [5, 6]. Its strength lies in handling high‐dimensional, non‐linear data characterized by complex interactions. This capability suggests ML may outperform traditional statistical models in tasks like disease diagnosis, risk stratification and decision support [7, 8]. However, for cachexia—a highly heterogeneous and dynamically evolving syndrome—there remains a lack of systematic ML studies based on large‐scale real‐world data that cover the entire clinical continuum from screening to prognosis [9, 10].

This study leverages the ‘Investigation on Nutrition Status and Clinical Outcome of Common Cancers’ (INSCOC) database to develop and validate a comprehensive ML system for predicting cancer cachexia progression [11, 12]. The system first classifies a patient's cachexia status at hospital admission and then, for those diagnosed, predicts survival to estimate both short‐term mortality risk and long‐term survival probability. This integrated tool aims to support the early warning, risk stratification and personalized management of cancer cachexia.

## 2 Method

### 2.1 Study Design and Population

**FIGURE 1:**
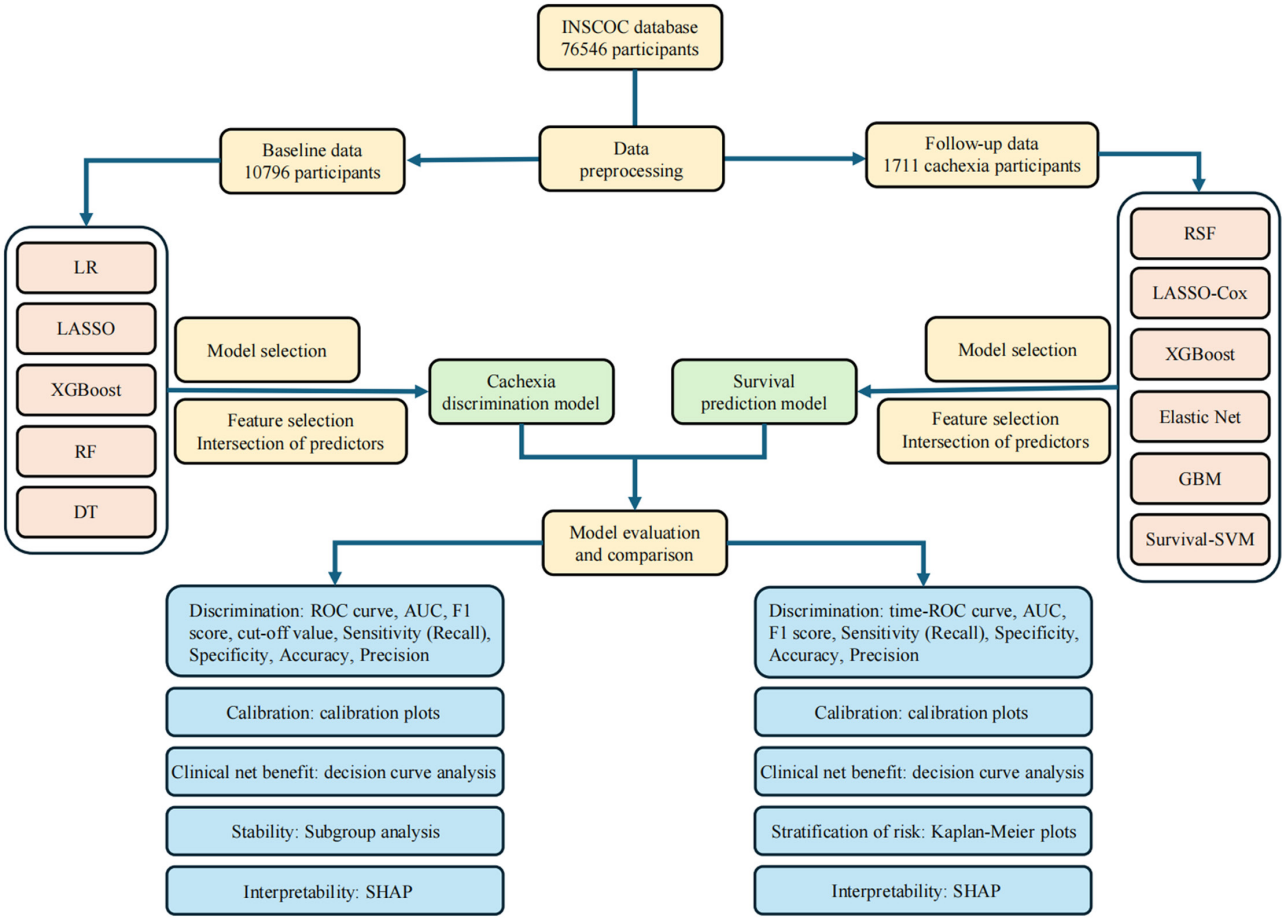
Analytical workflow for model development and validation. AUC, area under the (ROC) curve; DT, decision tree; GBM, gradient boosting machine; INSCOC, Investigation on Nutrition Status and its Clinical Outcome of Common Cancers; LASSO, least absolute shrinkage and selection operator; LR, logistic regression; RF, random forest; ROC, receiver operating characteristic; RSF, random survival forest; SHAP, SHapley additive explanations; SVM, support vector machine; XGBoost, extreme gradient boosting.

This multicentre, retrospective cohort study utilized data from the INSCOC project (registry number: ChiCTR1800020329), a database extensively employed in prior multicentre studies on cancer nutrition and prognosis. Detailed inclusion and exclusion criteria for the INSCOC project have been published previously [13, 14]. For the current analysis, we included adult patients (aged > 18 years) with cancer who were hospitalized for ≥ 48 h. Cancer cachexia was retrospectively diagnosed according to the 2011 international consensus proposed by Fearon et al. [15]. The diagnosis was made if a patient met one or more of the following criteria: involuntary weight loss > 5% within the past 6 months, or a body mass index (BMI) < 18.5 (based on Asian standards) accompanied by any degree of weight loss > 2%. Figure 1 shows the entire analysis workflow of the present study. A cohort including 886 oncology patients (the Fujian cohort), which was independent of the study population, was used for external validation.

The study protocol was approved by the Institutional Review Boards of all participating hospitals and conformed to the principles of the Declaration of Helsinki. Written informed consent for data use was obtained from all participants, in accordance with the ethical guidelines of the INSCOC project.

### 2.2 Variables

Baseline characteristics collected in this study included demographics, laboratory test results and anthropometric measurements. All personal identifiers were removed prior to analysis. Demographic data comprised sex, age and basic disease information such as tumour type and stage. Tumour stage was recorded as the stage at the time of the initial diagnosis. All laboratory tests were performed within 48 h of admission after at least 9 h of fasting. Blood tests included C‐reactive protein (CRP), blood cell count, white blood cell count, platelet count, neutrophil count, lymphocyte count, haemoglobin, alanine aminotransferase, aspartate aminotransferase, serum total protein, prealbumin, albumin, blood urea nitrogen, creatinine, total bilirubin, direct bilirubin, total cholesterol, blood glucose, triglycerides, high‐density lipoprotein (HDL) cholesterol and low‐density lipoprotein cholesterol. Anthropometric measurements included height, weight, mid–upper arm circumference, triceps skinfold thickness (TSF), handgrip strength of the non‐dominant hand, mid–upper arm muscle circumference, maximum calf circumference (CAC) and data on body‐weight change. Complete case analysis was adopted to handle missing data, and multiple imputations were not performed in this study.

Composite indices were calculated using the following formulas: BMI = weight (kg) / height (m)^2^; platelet‐to‐lymphocyte ratio (PLR) = platelets (×10^9^/L) / lymphocytes (×10^9^/L); neutrophil‐to‐lymphocyte ratio (NLR) = neutrophils (×10^9^/L) / lymphocytes (×10^9^/L); leukocyte‐to‐lymphocyte ratio (LLR) = leukocytes (×10^9^/L) / lymphocytes (×10^9^/L); prognostic nutritional index (PNI) = albumin (g/L) + 5 × lymphocytes (×10^9^/L); C‐reactive protein‐to‐albumin ratio (CAR) = CRP (mg/L) / albumin (g/L); and triglyceride‐glucose index (TyG) = ln [triglycerides (mg/dL) × glucose (mg/dL) / 2].

### 2.3 Training of ML Models

This study adopted a two‐stage modelling strategy, with stratified tenfold cross‐validation applied throughout model development to ensure stable generalization and reduce overfitting. The first stage built a cachexia discrimination model across the full cohort to identify cachexia‐related stable features. The second stage constructed a survival prediction model exclusively in cachexia patients to screen prognostic factors.

In the first stage, variable selection and importance evaluation were performed for the binary outcome of cachexia status (yes/no) using univariate logistic regression, multivariate logistic regression, least absolute shrinkage and selection operator (LASSO) logistic regression, eXtreme gradient boosting (XGBoost), random forest and decision tree. Variables selected by no fewer than three algorithms were defined as candidate predictors. Variance inflation factor (VIF) analysis was then conducted, and variables with VIF > 10 were excluded to eliminate multicollinearity, yielding the final feature set for the discrimination model. All variable screening, collinearity testing and model training were strictly implemented on cross‐validation training folds, whereas held‐out test folds were only used for final evaluation to avoid data leakage. Each training fold further adopted fivefold inner cross‐validation with early stopping to optimize boosting iterations, and model performance was quantified using cross‐validated AUC.

The second stage exclusively enrolled cachexia patients, with survival prediction performed based on time‐to‐event data derived from follow‐up status and time. Feature selection was conducted via random survival forest (RSF), LASSO‐Cox, XGBoost (survival framework), Elastic Net, gradient boosting machine (GBM) and survival‐support vector machine (SVM). Variables selected by at least four methods were regarded as prognostic features, which were further filtered by VIF testing before modelling. Similarly, stratified tenfold cross‐validation was implemented in the survival modelling pipeline with independent training and test partitioning to ensure unbiased evaluation. The final survival model was established using screened features and validated via cross‐validated predictive indices.

Furthermore, each model retained its top three most important features during training to avoid missing key predictors, regardless of their overall selection frequency. Mutually exclusive composite indicators were manually deduplicated to prevent redundant input. Consistent anti‐overfitting strategies, including early stopping, built‐in L1/L2 regularization and row/column subsampling, were applied across all models to improve robustness and biological plausibility.

### 2.4 Model Evaluation

For the cachexia discrimination model, we assessed performance and interpretability with respect to discrimination, calibration, clinical utility and robustness. Discrimination was quantified using the area under the receiver operating characteristic curve (AUC), as well as sensitivity (recall), specificity, accuracy, precision and the F1 score. The classification threshold was selected as prespecified, by maximizing the F1‐score. Clinical usefulness was evaluated using decision curve analysis (DCA) by estimating net benefit across a range of threshold probabilities, and calibration was examined with calibration curves comparing predicted and observed risks. Prespecified subgroup analyses were additionally conducted to examine model performance across clinically relevant strata. Model interpretability was investigated using Shapley additive explanations (SHAP) to quantify global feature contributions and to provide local, patient‐level explanations of individual predictions.

For the survival prediction model developed in patients with cachexia, we conducted a comprehensive evaluation on time‐to‐event data focusing on discrimination, calibration, clinical utility and risk stratification, with additional assessment of consistency across subgroups. Discrimination was primarily assessed using Harrell's concordance index (C‐index) and time‐dependent AUC at 3, 6, 9 and 12 months. As secondary time‐specific measures, we computed accuracy, precision, recall and F1 score at prespecified time horizons. Clinical utility was assessed using time‐dependent DCA to estimate net benefit across clinically relevant thresholds, and calibration was evaluated using time‐dependent calibration curves. For risk stratification, patients were categorized into high‐ and low‐risk groups according to model‐predicted risk, and between‐group survival differences were visualized using Kaplan–Meier curves and compared using the log‐rank test when appropriate. Model performance was further examined within clinically relevant subgroups to assess consistency and generalizability and to inform the selection of clinically actionable decision thresholds and risk stratification schemes.

Following model training, external validation was systematically performed to evaluate the model's discrimination and calibration performance.

### 2.5 Statistical Analysis

Continuous variables are presented as mean ± standard deviation or median [interquartile range]. Categorical variables are expressed as frequency (percentage). The chi‐square test was used for comparing categorical variables between groups. Model performance metrics were summarized as mean values with corresponding 95% confidence intervals (CI). The *t*‐test was employed for comparing continuous variables between groups. All tests were two‐sided, with statistical significance set at *p* < 0.05. All analyses were performed using R Studio (version 2025.09.2 Build 418).

## 3 Result

### 3.1 Baseline Characteristics

A total of 10 796 patients were included in this study, comprising 3995 (37.0%) with cachexia and 6801 (63.0%) without cachexia (Table 1, Figure S1). The overall cohort had a mean age of 59.8 ± 12.5 years, with 5951 (55.1%) males and 4845 (44.9%) females. Among the 5172 patients with available follow‐up data, the median follow‐up duration was 27.7 months (Table S1). In this follow‐up cohort including 1711 patients with cachexia, 586 events occurred. The 1‐year mortality rate was 20.8% (95%: CI 19.0%–22.9%). Baseline characteristics of the Fujian cohort are presented in Table S2.

**TABLE 1:** Baseline characteristics of the study population.

	Baseline	Cachexia	Non‐cachexia	*p*
*N*	10 796	3995	6801	
Sex				
Man	5951 (55.1%)	2421 (60.6%)	3530 (51.9%)	< 0.001
Women	4845 (44.9%)	1574 (39.4%)	3271 (48.1%)	
Age	59.8 [52.5, 66.4]	60.4 [53.2, 67.0]	59.5 [52.0, 65.9]	< 0.001
Smoking	4759 (44.1%)	1905 (47.7%)	2854 (42.0%)	< 0.001
Alcohol drinker	2193 (20.3%)	954 (23.9%)	1239 (18.2%)	< 0.001
Anticancer treatment				
Surgery	4954 (45.9%)	1877 (47%)	3077 (45.2%)	0.083
Chemotherapy	7011 (64.9%)	2582 (64.6%)	4429 (65.1%)	0.62
Radiotherapy	1070 (9.9%)	329 (8.2%)	741 (10.9%)	< 0.001
Others	8362 (77.5%)	3063 (76.7%)	5299 (77.9%)	0.142
Nutritional therapy				
Parenteral	801 (7.4%)	399 (10%)	402 (5.9%)	< 0.001
Enteral	1757 (16.3%)	925 (23.2%)	832 (12.2%)	< 0.001
None	8413 (77.9%)	2784 (69.7%)	5629 (82.8%)	< 0.001
Tumour stage				< 0.001
1	1061 (9.8%)	271 (6.8%)	790 (11.6%)	
2	1823 (16.9%)	589 (14.7%)	1234 (18.1%)	
3	3461 (32.1%)	1406 (35.2%)	2055 (30.2%)	
4	4451 (41.2%)	1729 (43.3%)	2722 (40.0%)	
PG‐SGA score	7.0 [2.0, 15.0]	15.0 [9.0, 22.0]	3.0 [1.0, 9.0]	< 0.001
Cachexia	3995 (37.0%)	—	—	
CRP	4.6 [2.2, 15.1]	5.9 [2.6, 25.0]	3.9 [2.0, 10.8]	< 0.001
RBC	4.2 [3.8, 4.6]	4.2 [3.7, 4.6]	4.3 [3.9, 4.7]	< 0.001
WBC	5.9 [4.6, 7.6]	6.0 [4.6, 7.9]	5.9 [4.6, 7.4]	< 0.001
PLT	222.0 [172.0, 281.0]	229.0 [176.4, 294.0]	219.0 [169.0, 273.0]	< 0.001
Neutrophils	3.6 [2.5, 5.1]	3.7 [2.5, 5.4]	3.5 [2.5, 4.9]	< 0.001
Lymphocyte	1.5 [1.1, 1.9]	1.4 [1.0, 1.8]	1.5 [1.1, 1.9]	< 0.001
HB	127.0 [113.0, 139.0]	123.0 [108.0, 136.0]	129.0 [116.0, 141.0]	< 0.001
ALT	17.9 [12.0, 27.7]	17.0 [11.3, 27.9]	18.0 [12.7, 27.6]	0.624
AST	21.2 [17.0, 28.2]	21.0 [16.5, 29.0]	21.4 [17.0, 28.0]	0.003
Total protein	67.9 [63.4, 72.3]	67.1 [62.4, 71.6]	68.3 [64.0, 72.7]	< 0.001
Albumin	39.1 [35.8, 42.2]	37.9 [34.4, 41.1]	39.7 [36.6, 42.6]	< 0.001
Prealbumin	0.2 [0.2, 0.3]	0.2 [0.1, 0.2]	0.2 [0.2, 0.3]	0.718
Total bilirubin	11.0 [8.3, 14.8]	11.3 [8.5, 15.4]	10.8 [8.1, 14.6]	< 0.001
Direct bilirubin	2.8 [2.0, 4.1]	3.0 [2.0, 4.3]	2.7 [2.0, 3.9]	< 0.001
Total cholesterol	4.6 [3.9, 5.3]	4.4 [3.8, 5.2]	4.6 [4.0, 5.4]	< 0.001
Triglyceride	1.3 [0.9, 1.8]	1.2 [0.9, 1.6]	1.3 [1.0, 1.9]	< 0.001
HDL	1.2 [1.0, 1.4]	1.1 [0.9, 1.4]	1.2 [1.0, 1.4]	< 0.001
LDL	2.8 [2.2, 3.4]	2.7 [2.2, 3.3]	2.8 [2.3, 3.4]	0.001
Glucose	5.3 [4.9, 6.0]	5.3 [4.8, 6.1]	5.4 [4.9, 6.0]	0.775
BUN	5.1 [4.1, 6.3]	5.0 [4.0, 6.3]	5.2 [4.2, 6.3]	0.954
Creatinine	64.9 [54.8, 76.9]	65.0 [54.8, 77.7]	64.6 [54.8, 76.2]	0.09
PLR	151.5 [109.7, 215.3]	162.9 [115.6, 235.3]	145.0 [106.5, 205.6]	< 0.001
NLR	2.4 [1.7, 3.8]	2.6 [1.7, 4.3]	2.3 [1.6, 3.5]	0.002
LLR	3.9 [3.0, 5.5]	4.2 [3.1, 6.2]	3.8 [3.0, 5.2]	0.112
PNI	46.7 [42.4, 50.5]	45.2 [40.7, 49.5]	47.5 [43.4, 51.2]	< 0.001
CAR	0.1 [0.1, 0.4]	0.2 [0.1, 0.7]	0.1 [0.0, 0.3]	< 0.001
TyG	8.6 [8.3, 9.0]	8.6 [8.2, 8.9]	8.7 [8.3, 9.1]	< 0.001
Height	165.0 [158.0, 170.0]	165.0 [159.0, 170.0]	164.0 [158.0, 170.0]	< 0.001
Weight	60.0 [54.0, 69.0]	57.0 [50.0, 65.0]	63.0 [56.0, 70.0]	< 0.001
BMI	22.6 [20.3, 24.9]	21.0 [19.0, 23.4]	23.4 [21.3, 25.6]	< 0.001
MAC	26.9 [24.5, 29.0]	25.5 [23.5, 27.9]	27.0 [25.0, 29.0]	< 0.001
TSF	15.0 [10.0, 20.0]	13.0 [9.0, 18.0]	16.0 [12.0, 22.0]	< 0.001
HGS	23.5 [17.9, 30.6]	22.7 [17.0, 30.0]	24.0 [18.3, 31.2]	< 0.001
MAMC	21.6 [19.7, 23.7]	21.2 [19.1, 23.2]	22.0 [20.1, 24.0]	< 0.001
CAC	34.0 [32.0, 36.5]	33.0 [30.9, 35.0]	35.0 [32.5, 37.0]	< 0.001

*Note:* Other anticancer treatments include endocrine therapy, targeted therapy, immunotherapy, interventional embolization, radiofrequency ablation, cryotherapy and hyperthermia. Continuous variables are presented as median [interquartile range]. Categorical variables are expressed as frequency (percentage). Classification variables between groups using chi‐square test. Continuous variables were compared between groups using *t*‐test.

Abbreviations: ALT, alanine aminotransferase; AST, aspartate aminotransferase; BMI, body mass index; BUN, blood urea nitrogen; CAC, calf circumference; CAR, C‐reactive protein‐to‐albumin ratio; CRP, C‐reactive protein; HB, haemoglobin; HDL, high‐density lipoprotein; HGS, handgrip strength; LDL, low‐density lipoprotein; LLR, leukocyte‐to‐lymphocyte ratio; MAC, mid‐arm circumference; MAMC, mid‐arm muscle circumference; NLR, neutrophil‐to‐lymphocyte ratio; PG‐SGA, patient‐generated subjective global assessment; PLR, platelet‐to‐lymphocyte ratio; PLT, platelet count; PNI, prognostic nutritional index; RBC, red blood cell count; SII, systemic immune‐inflammation index; TSF, triceps skinfold thickness; TyG, triglyceride‐glucose index; WBC, white blood cell count.

Cancer types included pancreatic cancer, cholangiocarcinoma, gastric cancer, oesophageal cancer, colorectal cancer, gastrointestinal stromal tumour, ovarian cancer, malignant brain tumour, cervical cancer, liver cancer, prostate cancer, lung cancer, nasopharyngeal carcinoma, endometrial cancer, bladder cancer, breast cancer and other malignancies. The distribution of cancer types and the prevalence of cachexia within each type are detailed in Table S3. Lung cancer was the most common malignancy (*n* = 3629), whereas pancreatic cancer exhibited the highest prevalence of cachexia (63.41%).

### 3.2 Variable Selection and Model Training

Based on whether the prevalence of cachexia was ≥ 40%, the overall population was divided into a high‐prevalence group and a low‐prevalence group, and cachexia identification models were constructed for each group, respectively (Table S4). Additionally, a survival prediction model was developed based on cachexia patients with follow‐up data.

Variable selection was performed first. Except for variables directly related to the diagnosis of cachexia, all baseline characteristics were input into ML models to assess variable importance. The top 10 most important variables from each model were selected, and their frequency of occurrence across all models was counted (Tables S5–S7). Based on the selection frequency, variables were ranked to determine the candidate feature set. Following the exclusion of multicollinear variables using VIF testing, the final variables incorporated into each model are listed below. The cachexia classification model for the high‐prevalence group included the variables detailed in Table S8; the model for the low‐prevalence group comprised those in Table S9. For the survival prediction model in cachexia patients, the selected variables are presented in Table S10.

### 3.3 Model Validation of Cachexia Discrimination Model

**FIGURE 2:**
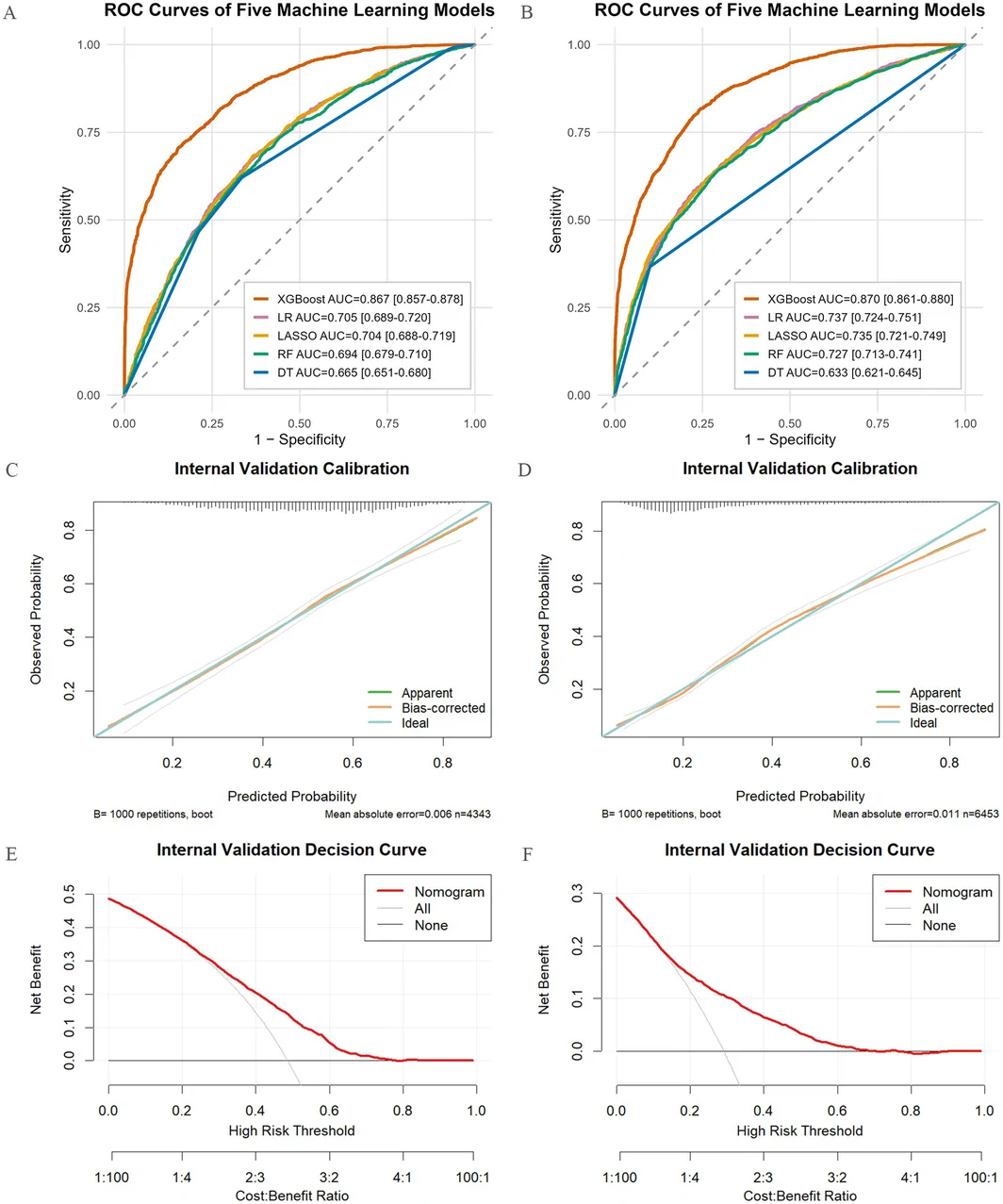
Discriminative ability, calibration and clinical utility of the cachexia discrimination model (based on XGBoost algorithm) in the high‐ and low‐prevalence groups. (A) ROC curves of five machine learning models for cachexia classification in the high‐prevalence group. (B) ROC curves of five machine learning models for cachexia classification in the low‐prevalence group. (C) Calibration plot of the cachexia classification model in the high‐prevalence group. (D) Calibration plot of the cachexia classification model in the low‐prevalence group. (E) Decision curve analysis of the cachexia classification model in the high‐prevalence group. (F) Decision curve analysis of the cachexia classification model in the low‐prevalence group. AUC, area under the (ROC) curve; DT, decision tree; LASSO, least absolute shrinkage and selection operator; LR, logistic regression; RF, random forest; ROC, receiver operating characteristic; XGBoost, extreme gradient boosting.

Table S11 compares the predictive performance metrics of different ML algorithms under different combinations of variables. The XGBoost model demonstrated good discriminative performance in both the high‐ and low‐prevalence groups (Table 2). In the high‐prevalence group, the XGBoost model achieved an AUC of 0.867 (95% CI: 0.857–0.878), indicating strong discrimination ability (Figure 2A). At the optimal cut‐off value of 0.541, the sensitivity (recall) was 0.706, and the specificity was 0.846, with an overall accuracy of 0.778. In addition, the precision of the XGBoost model was 0.813, and the F1 score was 0.756, suggesting favourable precision and balanced predictive performance in the high‐prevalence population. In the low‐prevalence group, the XGBoost model yielded an AUC of 0.870, indicating stable and high discriminative ability (Figure 2B). Using an optimal cut‐off value of 0.279, the sensitivity was 0.817, and the specificity was 0.756, with an accuracy of 0.774. Furthermore, the precision was 0.579, and the F1 score was 0.678, indicating that the XGBoost model maintained a relatively balanced performance while achieving a high recall in the low‐prevalence population.

**TABLE 2:** Performance comparison of cachexia classification models built with different machine learning algorithms.

Model	Multivariate LR	LASSO	XGBoost	RF	DT
High‐prevalence group					
AUC	0.705 [0.689, 0.720]	0.704 [0.688, 0.719]	0.867 [0.857, 0.878]	0.694 [0.679, 0.710]	0.665 [0.651, 0.680]
Cut‐off value	0.470	0.480	0.541	0.463	0.457
Sensitivity (recall)	0.695 [0.563, 0.768]	0.682 [0.622, 0.773]	0.706 [0.680, 0.834]	0.699 [0.586, 0.763]	0.621 [0.600, 0.641]
Specificity	0.615 [0.541, 0.745]	0.634 [0.541, 0.697]	0.846 [0.721, 0.872]	0.595 [0.532, 0.710]	0.665 [0.645, 0.685]
Accuracy	0.654 [0.644, 0.670]	0.657 [0.644, 0.671]	0.778 [0.767, 0.791]	0.646 [0.636, 0.663]	0.644 [0.629, 0.657]
Precision	0.631 [0.610, 0.685]	0.639 [0.610, 0.664]	0.813 [0.734, 0.836]	0.621 [0.599, 0.664]	0.638 [0.616, 0.659]
F1 score	0.662 [0.611, 0.683]	0.660 [0.636, 0.687]	0.756 [0.744, 0.787]	0.658 [0.615, 0.682]	0.629 [0.611, 0.646]
Low‐prevalence group					
AUC	0.737 [0.724, 0.751]	0.735 [0.721, 0.749]	0.870 [0.861, 0.880]	0.727 [0.713, 0.741]	0.633 [0.621, 0.645]
Cut‐off value	0.340	0.319	0.279	0.301	0.413
Sensitivity (recall)	0.583 [0.559, 0.748]	0.619 [0.568, 0.690]	0.817 [0.776, 0.838]	0.639 [0.598, 0.662]	0.366 [0.345, 0.389]
Specificity	0.775 [0.607, 0.797]	0.735 [0.674, 0.785]	0.756 [0.741, 0.795]	0.708 [0.694, 0.746]	0.900 [0.892, 0.908]
Accuracy	0.719 [0.649, 0.732]	0.702 [0.673, 0.725]	0.774 [0.764, 0.794]	0.688 [0.677, 0.709]	0.744 [0.734, 0.755]
Precision	0.516 [0.439, 0.540]	0.491 [0.455, 0.528]	0.579 [0.561, 0.616]	0.474 [0.457, 0.504]	0.601 [0.574, 0.630]
F1 score	0.547 [0.534, 0.570]	0.547 [0.532, 0.569]	0.678 [0.664, 0.697]	0.544 [0.528, 0.564]	0.455 [0.434, 0.478]

Abbreviations: AUC, area under the (receiver operating characteristic) curve; DT, decision tree; LASSO, least absolute shrinkage and selection operator; LR, logistic regression; RF, random forest; XGBoost, eXtreme gradient boosting.

In the internal validation, calibration curves and DCA were used to evaluate the agreement between predicted and observed risks and the potential clinical utility of the XGBoost model in both prevalence groups. In the high‐prevalence group, the calibration curve showed good agreement between the predicted probabilities and the observed outcomes, with the apparent and bias‐corrected curves closely overlapping the ideal reference line (Figure 2C). DCA demonstrated that the XGBoost model yielded a higher net benefit than the treat‐all and treat‐none strategies across a wide range of threshold probabilities, suggesting potential clinical utility in risk stratification within the high‐prevalence population (Figure 2E). In the low‐prevalence group, the calibration curve also indicated acceptable agreement between predicted and observed probabilities, with the apparent and bias‐corrected curves remaining close to the ideal line (Figure 2D). DCA showed that the XGBoost model provided a positive net benefit over the treat‐all and treat‐none strategies within a limited range of lower to intermediate threshold probabilities, whereas the net benefit gradually approached zero at higher threshold values (Figure 2F). These results suggest that the XGBoost model demonstrates good calibration and potential clinical usefulness in both high‐ and low‐prevalence populations, with more robust net benefit observed in the high‐prevalence group. The XGBoost model showed robust and balanced predictive performance across all clinical and demographic subgroups in both high‐ and low‐prevalence groups (Table S12), indicating considerable generalizability and clinical applicability.

**FIGURE 3:**
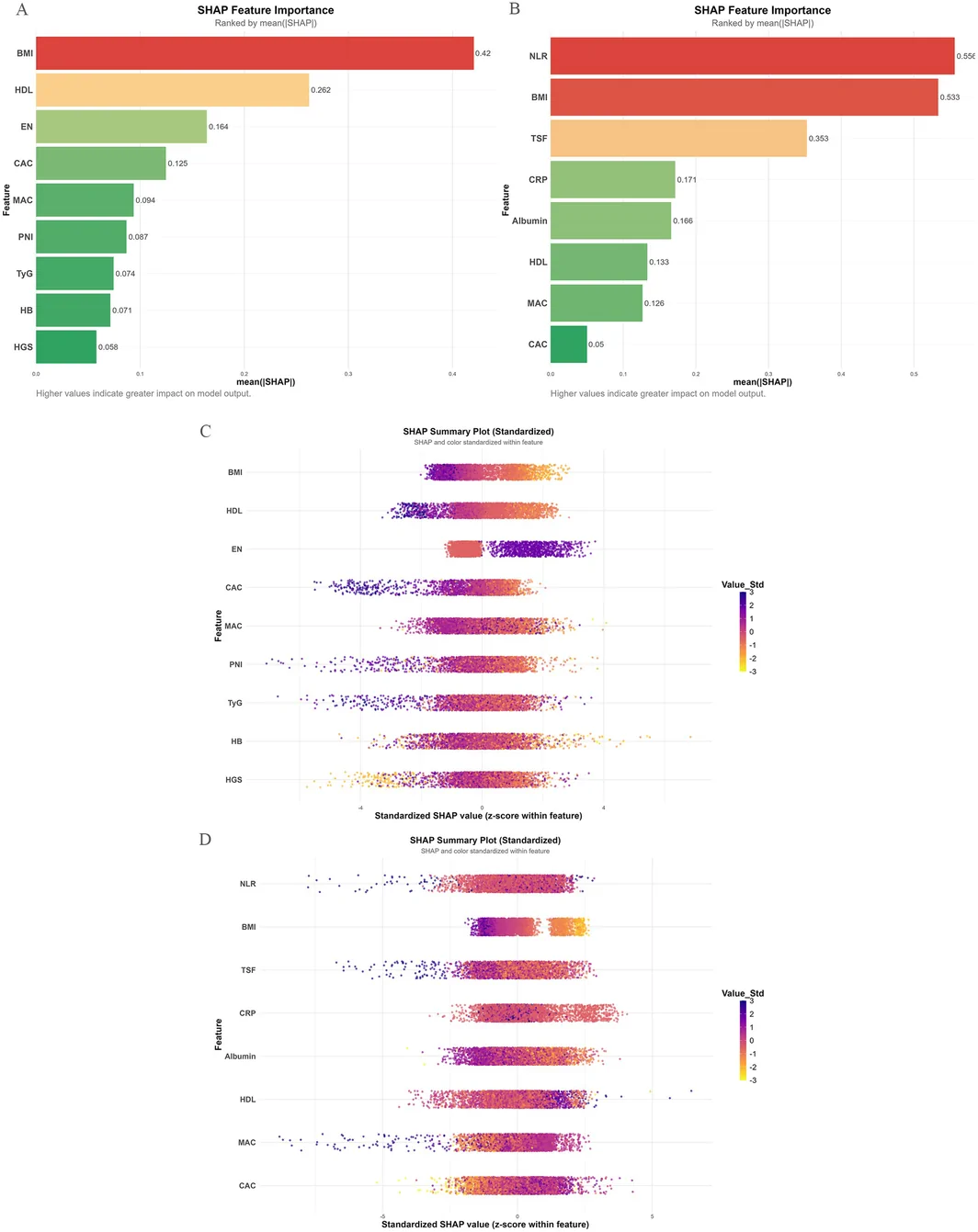
Feature importance and interpretation of the XGBoost‐based cachexia classification model in the high‐ and low‐prevalence groups. (A) Feature importance ranking based on mean |SHAP value| in the high‐prevalence group. (B) Feature importance ranking based on mean |SHAP value| in the low‐prevalence group. (C) Standardized SHAP summary plot (z‐score normalized) for the high‐prevalence group. (D) Standardized SHAP summary plot (z‐score normalized) for the low‐prevalence group. BMI, body mass index; CAC, calf circumference; CRP, C‐reactive protein; EN, enteral nutrition; HB, haemoglobin; HDL, high‐density lipoprotein; HGS, hand grip strength; MAC, mid‐arm circumference; NLR, neutrophil‐to‐lymphocyte ratio; PNI, prognostic nutritional index; SHAP, SHapley additive explanations; TSF, triceps skinfold thickness; TyG, triglyceride‐glucose index; XGBoost, extreme gradient boosting.

SHAP analysis was performed to interpret the XGBoost model and to identify the key features contributing to cachexia risk prediction in both the high‐ and low‐prevalence groups (Figures S2–S5). In the high‐prevalence group, feature importance ranking based on mean absolute SHAP values indicated that BMI was the most influential predictor, followed by HDL, enteral nutrition (EN) and CAC (Figure 3A). The SHAP summary plot demonstrated that lower BMI and HDL values generally pushed the model output toward higher cachexia risk, whereas higher values were associated with reduced risk (Figure 3C). Individual‐level SHAP force plots further illustrated that nutritional and body composition–related features played a dominant role in driving model predictions toward cachexia or non‐cachexia outcomes in this group (Figure S6A,C). In contrast, in the low‐prevalence group, NLR emerged as the most important feature, followed by BMI and TSF (Figure 3B). Inflammatory markers such as NLR and CRP showed strong positive contributions to increased cachexia risk, whereas higher BMI, TSF and albumin levels were generally associated with lower predicted risk. The SHAP summary plot revealed greater heterogeneity in feature contributions at the individual level (Figure 3D), and representative SHAP force plots confirmed that inflammation‐related variables exerted a stronger influence on model predictions in the low‐prevalence population (Figure S6B,D). Overall, SHAP analysis highlighted distinct feature contribution patterns between the two prevalence groups, with nutritional and body composition indicators predominating in the high‐prevalence group and inflammation‐related features playing a more prominent role in the low‐prevalence group, thereby enhancing the interpretability of the XGBoost model across different clinical contexts.

The Fujian cohort (*n* = 886) was enrolled as the external validation set. In the high‐prevalence group (*n* = 626), the original model yielded an AUC of 0.693 (95% CI: 0.652–0.735) and a Brier score of 0.222; after transfer learning optimization, the discriminative ability and calibration were substantially improved, with an AUC of 0.744 and a Brier score of 0.204. For the low‐prevalence group (*n* = 260), the model achieved an AUC of 0.694 (95% CI: 0.613–0.776) and a Brier score of 0.175. Given the limited sample size of the low‐prevalence group, further optimization and adjustment were not performed in the present study.

### 3.4 Model Validation of Survival Prediction Model

**FIGURE 4:**
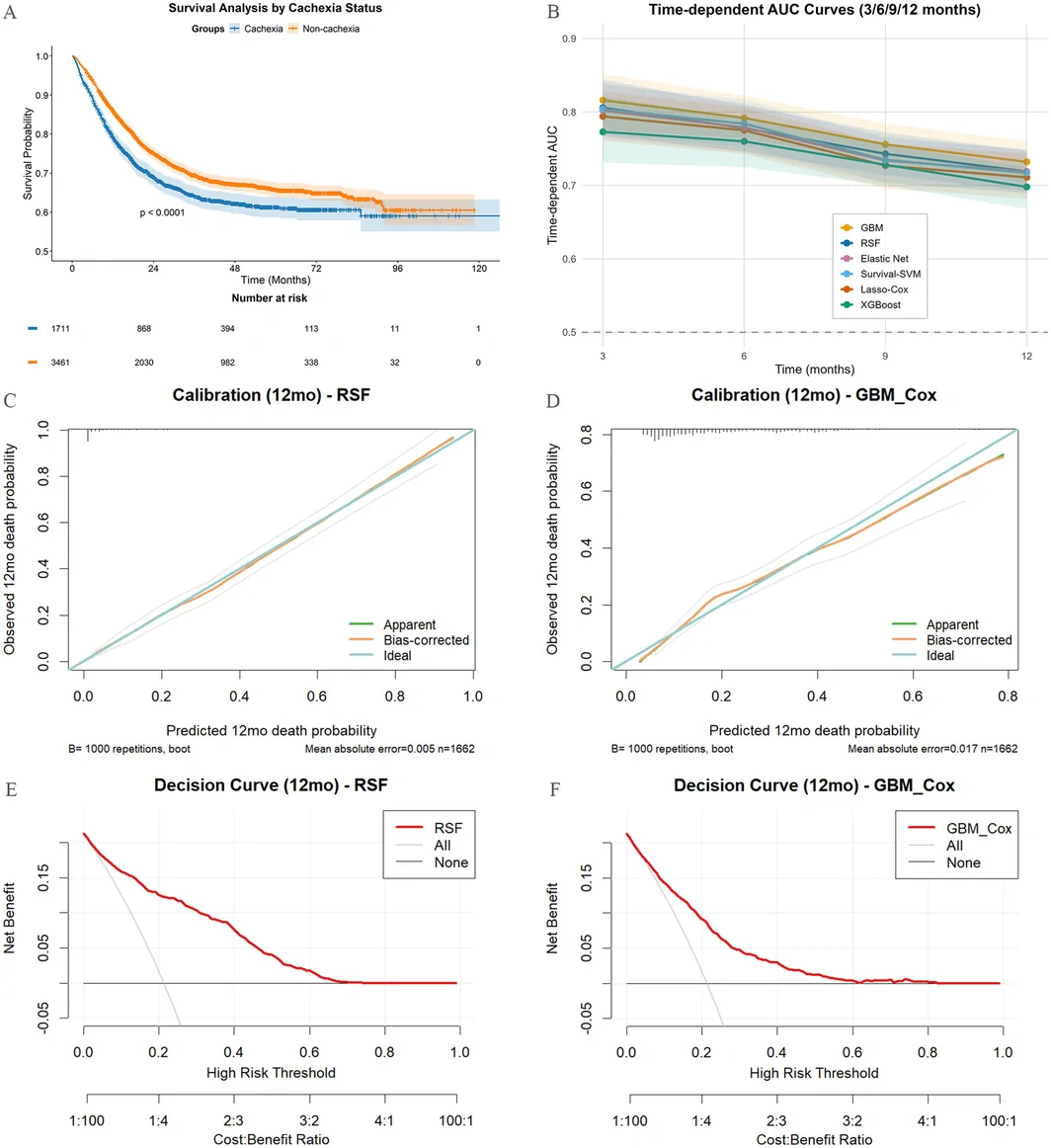
Performance evaluation and clinical utility of the survival prediction models for cachexia patients. (A) Kaplan–Meier survival curves stratified by cachexia status. The hazard ratio (HR) for non‐cachexia versus cachexia (reference) was 0.793 (95% CI: 0.716–0.877, *p* < 0.0001). The total number of events was 1614 (31.21%). (B) Time‐dependent ROC curves of six machine learning models for survival prediction. (C) Calibration plot of the RSF‐based model. (D) Calibration plot of the GBM‐based model. (E) Decision curve analysis of the RSF‐based model. (F) Decision curve analysis of the GBM‐based model. AUC, area under the (ROC) curve; GBM, gradient boosting machine; ROC, receiver operating characteristic; RSF, random survival forest; SVM, support vector machine; XGBoost, extreme gradient boosting.

Figure 4A shows that patients with cachexia had significantly worse overall survival than those without cachexia throughout follow‐up, with an early and persistent separation of the Kaplan–Meier curves, highlighting the strong prognostic impact of cachexia status. Among the various survival prediction models constructed based on the cachexia patient cohort, the RSF and GBM demonstrated significantly superior overall predictive performance compared to other models (Table 3). Specifically, the C‐index values for RSF and GBM, reflecting their discriminative ability, were 0.680 and 0.685, respectively, indicating comparable performance. In terms of time‐dependent AUC at 3, 6, 9 and 12 months, both models consistently exhibited excellent performance, demonstrating strong temporal discriminative power relative to other models (Figure 4B). From the perspective of key performance metrics, the classification performance of RSF and GBM at 12 months was particularly outstanding. Both models achieved high recall (RSF: 0.976, GBM: 0.978) alongside high precision (RSF: 0.804, GBM: 0.805), resulting in F1‐scores exceeding 0.88, which were significantly higher than those of other models.

**TABLE 3:** Performance comparison of survival prediction models for cachexia patients built with different machine learning algorithms.

	RSF	LASSO‐Cox	XGBoost	Elastic Net	GBM	Survival‐SVM
C index	0.680	0.683	0.666	0.684	0.685	0.681
AUC‐3m	0.800 [0.762, 0.839]	0.794 [0.761, 0.828]	0.768 [0.724, 0.814]	0.802 [0.765, 0.840]	0.816 [0.776, 0.852]	0.804 [0.769, 0.837]
AUC‐6 m	0.777 [0.746, 0.808]	0.775 [0.743, 0.806]	0.752 [0.717, 0.785]	0.779 [0.746, 0.812]	0.792 [0.764, 0.822]	0.784 [0.753, 0.817]
AUC‐9 m	0.741 [0.713, 0.770]	0.727 [0.696, 0.757]	0.721 [0.690, 0.752]	0.734 [0.703, 0.765]	0.756 [0.732, 0.784]	0.735 [0.704, 0.766]
AUC‐12 m	0.716 [0.689, 0.744]	0.711 [0.682, 0.743]	0.703 [0.672, 0.732]	0.719 [0.690, 0.749]	0.732 [0.703, 0.760]	0.717 [0.689, 0.749]
Accuracy‐12 m	0.793 [0.771, 0.813]	0.765 [0.744, 0.784]	0.782 [0.762, 0.802]	0.792 [0.773, 0.810]	0.796 [0.776, 0.816]	0.780 [0.762, 0.800]
Precision‐12 m	0.804 [0.774, 0.810]	0.403 [0.345, 0.462]	0.426 [0.347, 0.504]	0.430 [0.167, 0.710]	0.805 [0.788, 0.826]	0.321 [0.148, 0.500]
Recall‐12 m	0.976 [0.773, 0.812]	0.282 [0.237, 0.330]	0.163 [0.127, 0.203]	0.017 [0.006, 0.032]	0.978 [0.970, 0.986]	0.028 [0.011, 0.047]
F1 score‐12 m	0.882 [0.773, 0.811]	0.332 [0.285, 0.380]	0.235 [0.189, 0.285]	0.033 [0.011, 0.061]	0.884 [0.871, 0.896]	0.052 [0.021, 0.084]

Abbreviations: AUC, area under the (receiver operating characteristic) curve; GBM, gradient boosting machine; LASSO, least absolute shrinkage and selection operator; RSF, random survival forest; SVM, support vector machine; XGBoost, eXtreme gradient boosting.

In the comparison of calibration curves, the RSF model outperformed the GBM model, demonstrating greater stability in calibration capability (Figure 4C,D). After bias correction, its calibration curve showed a high degree of alignment with the ideal reference line, indicating superior consistency between predicted probabilities and actual observed outcomes. Based on its better calibration performance and excellent predictive discrimination ability, the RSF model was established as the most advantageous survival prediction model in this study. In DCA, the RSF model exhibited higher clinical net benefit at all time points, particularly at lower risk thresholds, effectively identifying high‐risk patients and providing more reliable support for clinical decision‐making (Figure 4E,F). Although the GBM model also demonstrated favourable net benefit at multiple time points, its overall decision curve performance remained slightly inferior to that of the RSF model. Although both the RSF and GBM models showed good predictive performance, the RSF model was ultimately selected as the optimal survival prediction model due to its comprehensive advantages in calibration consistency and clinical decision support (Figures S7 and S8).

In the RSF survival prediction model, key prognostic variables were further identified and interpreted through feature importance analysis and partial dependence plots (PDPs) (Figure S9). The variable importance measure (VIMP) indicated that tumour–node–metastasis (TNM) stage had the highest importance (VIMP = 0.177), followed by albumin (0.067) and prealbumin (0.049), suggesting that markers related to tumour progression and nutritional status possess strong predictive power for prognosis. In contrast, CRP and HDL showed relatively lower importance (0.044 and 0.033, respectively). The results from the PDPs were consistent with the aforementioned importance ranking. The model demonstrated robust risk stratification capability. Kaplan–Meier survival curves revealed significant differences in survival rates among groups stratified by each clinical indicator (Figure S10). Specifically, poorer survival outcomes were significantly associated with advanced TNM stage, lower levels of albumin and prealbumin, lower HDL levels and higher levels of CRP and neutrophils. These findings indicate that the variables selected by the model can effectively discriminate patient risk and possess clear prognostic value.

We developed an interactive web‐based tool using R Shiny to implement the cachexia diagnostic and survival prediction models (https://sinkhuang.shinyapps.io/cachexia/). The interface is user‐friendly, with dynamic variable inputs and graphical outputs, facilitating bedside risk stratification and clinical decision‐making (Figure S11).

## 4 Discussion

Based on the INSCOC multicentre real‐world database, this study developed and validated a two‐stage ML prediction system for cancer cachexia, designed for cachexia identification and survival risk assessment in diagnosed patients. The results demonstrated that the established models exhibited good discrimination, calibration and potential clinical utility across populations with different cachexia prevalence rates and were able to untangle heterogeneous patterns of cachexia‐related risk factors under different clinical contexts. These findings provide new data‐driven evidence for the precise identification, risk stratification and individualized management of cancer cachexia.

In the cachexia identification stage, the XGBoost model achieved comparable discrimination in high‐ and low‐prevalence cohorts but displayed distinct core predictive features, revealing clinical heterogeneity of cancer cachexia. In the high‐prevalence group, BMI, HDL and anthropometric measures dominated predictions. Decreased HDL reflects inflammation‐mediated lipid dysregulation central to cachexia‐related energy imbalance [16, 17, 18, 19]. Consistent with Table 1's intergroup baseline differences, cachexia patients had lower BMI, TSF, mid‐arm circumference, CAC, handgrip strength and albumin, representing global loss of fat and muscle tissue, which matches the canonical cachexia phenotype characterized by progressive lean mass wasting [20, 21, 22].

By contrast, inflammatory markers NLR and CRP carried higher predictive weight in the low‐prevalence group, whereas nutritional indicators contributed less. Systemic inflammation precedes visible weight and muscle loss in subclinical, early cachexia, enabling earlier screening [23, 24, 25, 26]. SHAP analyses validated these divergent feature weights across subgroups and improved the model's biological interpretability.

Collectively, our results indicate cancer cachexia is a dynamically progressive, stage‐heterogeneous syndrome rather than a fixed clinical state [15, 27]. The shift from inflammation‐driven risk signatures in low‐prevalence populations to nutritional/body composition depletion signals in high‐prevalence cohorts suggests a disease trajectory from inflammatory dysregulation to overt metabolic and muscular impairment [28, 29].

Among the survival prediction models for patients with cachexia, the RSF demonstrated comprehensive advantages in discrimination, calibration and clinical net benefit. Key prognostic variables identified by the model included TNM stage, albumin, prealbumin and inflammation‐related indicators, suggesting that the prognosis of cachexia patients is determined by the combined effects of tumour burden, nutritional depletion and systemic inflammation, rather than by any single factor [30, 31, 32, 33, 34]. PDPs and risk stratification analysis further confirmed that these variables effectively distinguish patients with different levels of prognostic risk, and the findings were highly consistent with previous clinical and biological research, thereby enhancing the credibility of the model predictions [35, 36].

From a clinical implementation perspective, the proposed prediction framework can be integrated into routine oncology and nutritional screening workflows as follows: Upon hospital admission, patient data—including height, weight, laboratory tests and anthropometric measurements—are collected and automatically processed by the system, which generates two outputs: cachexia classification and survival risk stratification. These outputs inform two distinct clinical decisions—whether to initiate nutritional intervention and how intensive that intervention should be. The cachexia identification model can be applied for early screening and risk warning across different clinical contexts, particularly for identifying high‐risk individuals among patients who have not yet developed overt weight loss [37, 38]. The survival prediction model can assist in risk stratification for patients already diagnosed with cachexia, providing decision support for nutritional support, anti‐inflammatory therapy and comprehensive management strategies [39, 40]. Notably, all variables required by the models are routine clinical and laboratory indicators, ensuring high accessibility and facilitating widespread adoption.

This study has several methodological strengths. First, it is based on large‐scale, multicentre real‐world data, which enhances the robustness and clinical relevance of the findings. Second, the two‐stage modelling strategy optimizes cachexia identification and prognosis assessment separately, avoiding the dilution of important risk signals that may occur in mixed‐population modelling. Third, multiple ML and survival analysis methods were integrated for variable selection, and stable features were identified through consensus criteria, improving model generalizability. Fourth, the evaluation framework is comprehensive, covering discrimination, calibration, DCA and subgroup consistency. This is complemented by SHAP‐ and PDP‐based model interpretation, which strengthens the clinical interpretability and practical utility of the prediction framework. Finally, the model's generalizability was externally validated in an independent Fujian cohort, confirming stable predictive performance across different populations. Nonetheless, before large‐scale clinical deployment, appropriate adaptive adjustments based on local clinical characteristics are still recommended.

This study has several inherent limitations. First, its retrospective nature introduces unavoidable selection bias and residual confounding from unrecorded clinical factors. Second, while the model achieved acceptable predictive performance in the external Fujian validation cohort, subgroup analyses for low cachexia‐prevalence tumour types were constrained by limited sample size, which also partially accounts for the moderate performance drop observed across external sites. Additional interhospital disparities contributing to cross‐cohort performance variability include divergent patient demographic distributions, heterogeneous case mixes and inconsistent measurement standards across participating centres. Third, our cachexia diagnostic criteria omitted skeletal muscle depletion evaluation, which may miss patients presenting with isolated muscle wasting without substantial weight loss. Fourth, the model only incorporated static admission baseline variables and lacked longitudinal time‐varying clinical markers, restricting its capacity to track progressive alterations throughout cachexia development. These caveats underscore the necessity of larger, more heterogeneous cohorts for external validation prior to broad clinical implementation. Nevertheless, the performance gain achieved via transfer learning confirms the model's adaptability to independent patient populations. Moving forward, we plan to adopt prospective multicentre designs, integrate objective muscle mass quantification and construct dynamic predictive models to refine and upgrade the present risk prediction framework.

## 5 Conclusion

Based on a large multicentre real‐world cohort, this study developed and externally validated an explainable two‐stage ML framework for early identification and prognostic stratification of cancer cachexia. The framework achieved stable predictive performance in both high‐ and low‐prevalence populations and revealed distinct context‐dependent risk patterns of cachexia. Good generalizability of the models was confirmed in an independent external cohort. This well‐validated, clinically accessible framework can facilitate early recognition and individualized management of cancer cachexia in routine practice.

## Funding

This study was supported by the National Key Research and Development Program of China (No. 2022YFC2009600).

## Ethics Statement

All human studies referenced above were approved by the local Institutional Review Board and have been performed in accordance with the Declaration of Helsinki and its amendments. All participants provided informed consent prior to inclusion in this study, and care has been taken to reduce the likelihood of any details that might disclose participant identities.

## Conflicts of Interest

The authors declare no conflicts of interest.

## Supporting information


**Table S1:** The baseline data of the population were followed up.
**Table S2:** Baseline characteristics of the Fujian external validation cohort.
**Table S3:** The distribution of different cancer types and the incidence rate of cachexia in this study.
**Table S4:** Baseline data for the high‐ and low‐prevalence groups.
**Table S5:** Feature importance ranking for cachexia classification in the high‐prevalence group.
**Table S6:** Feature importance ranking for cachexia classification in the high‐prevalence group.
**Table S7:** Feature importance ranking for the survival prediction model in cachexia patients.
**Table S8:** Variables selected for the prediction model in the high‐prevalence group after feature screening.
**Table S9:** Variables selected for the prediction model in the low‐prevalence group after feature screening.
**Table S10:** Variables selected for the survival prediction model in cachexia patients.
**Table S11:** Predictive performance metrics of different machine learning algorithms under different variables.
**Table S12:** Subgroup analysis of the XGBoost‐based cachexia classification model in the high‐ and low‐prevalence groups.

**Figure S1:** Flowchart of participant selection and data preprocessing from the INSCOC database. INSCOC, Investigation on Nutrition Status and Clinical Outcome of Common Cancers.

**Figure S2:** SHAP dependence plots for key features in the XGBoost‐based cachexia classification model (high‐prevalence group). (A) Dependence of SHAP values on BMI. (B) Dependence of SHAP values on HDL. (C) Dependence of SHAP values on EN. (D) Dependence of SHAP values on CAC. (E) Dependence of SHAP values on MAC. BMI, body mass index; CAC, calf circumference; EN, enteral nutrition; HDL, high‐density lipoprotein; MAC, mid‐arm circumference; SHAP, SHapley additive explanations; XGBoost, eXtreme gradient boosting.

**Figure S3:** SHAP dependence plots for key features in the XGBoost‐based cachexia classification model (high‐prevalence group). (A) Dependence of SHAP values on PNI. (B) Dependence of SHAP values on HDL. (C) Dependence of SHAP values on TyG. (D) Dependence of SHAP values on HB. (E) Dependence of SHAP values on HGS. HB, haemoglobin; HGS, hand grip strength; PNI, prognostic nutritional index; SHAP, SHapley additive explanations; TyG, triglyceride‐glucose index; XGBoost, eXtreme gradient boosting.

**Figure S4:** SHAP dependence plots for key features in the XGBoost‐based cachexia classification model (low‐prevalence group). (A) Dependence of SHAP values on NLR. (B) Dependence of SHAP values on BMI. (C) Dependence of SHAP values on TSF. (D) Dependence of SHAP values on CRP. BMI, body mass index; CRP, C‐reactive protein; NLR, neutrophil‐to‐lymphocyte ratio; SHAP, SHapley additive explanations; TSF, triceps skinfold thickness; XGBoost, eXtreme gradient boosting.

**Figure S5:** SHAP dependence plots for key features in the XGBoost‐based cachexia classification model (low‐prevalence group). (A) Dependence of SHAP values on albumin. (B) Dependence of SHAP values on HDL. (C) Dependence of SHAP values on MAC. (D) Dependence of SHAP values on CAC. CAC, calf circumference; HDL, high‐density lipoprotein; MAC, mid‐arm circumference; SHAP, SHapley additive explanations; XGBoost, eXtreme gradient boosting.

**Figure S6:** Case‐level interpretation of the XGBoost classifier using SHAP force plots in the high‐ and low‐prevalence groups. (A) A cachexia case from the high‐prevalence group. (B) A cachexia case from the low‐prevalence group. (C) A non‐cachexia case from the high‐prevalence group. (D) A non‐cachexia case from the low‐prevalence group. BMI, body mass index; CAC, calf circumference; CRP, C‐reactive protein; EN, enteral nutrition; HB, haemoglobin; HDL, high‐density lipoprotein; HGS, hand grip strength; MAC, mid‐arm circumference; NLR, neutrophil‐to‐lymphocyte ratio; PNI, prognostic nutritional index; SHAP, SHapley additive explanations; TSF, triceps skinfold thickness; TyG, triglyceride‐glucose index.

**Figure S7:** Calibration and clinical utility of the RSF survival prediction model across different time points. (A) Calibration plot at 3 months. (B) Decision curve analysis at 3 months. (C) Calibration plot at 6 months. (D) Decision curve analysis at 6 months. (E) Calibration plot at 9 months. (F) Decision curve analysis at 9 months. RSF, random survival forest.

**Figure S8:** Calibration and clinical utility of the GBM survival prediction model across different time points. (A) Calibration plot at 3 months. (B) Decision curve analysis at 3 months. (C) Calibration plot at 6 months. (D) Decision curve analysis at 6 months. (E) Calibration plot at 9 months. (F) Decision curve analysis at 9 months. RSF, random survival forest. GBM, gradient boosting machine.

**Figure S9:** Interpretability of the survival prediction model: feature importance and partial dependence plots. (A) Variable importance ranking of the random survival forest (RSF)‐based model. (B–G) Partial dependence plots showing the marginal effect on the predicted 12‐month survival probability for: (B) TNM stage, (C) albumin, (D) prealbumin, (E) HDL, (F) CRP and (G) neutrophil count. CRP, C‐reactive protein; HDL, high‐density lipoprotein; Neu, neutrophil; RSF, random survival forest; TNM, tumour, node, metastasis (staging system); VIMP, variable importance.

**Figuure S10.** Prognostic stratification using Kaplan–Meier survival analysis according to clinical variables. (A) Stratified by TNM stage. (B–F) Stratified by tertiles (low, medium and high) of (B) albumin, (C) prealbumin, (D) HDL, (E) CRP and (F) neutrophil count. The hazard ratios (HRs), 95% confidence intervals (CIs) and *p*‐values were derived from univariate Cox regression analyses with the reference groups as indicated. (A) TNM stage (vs. Stage I): Stage II, HR = 1.695 (95% CI: 0.977–2.939, *p* = 0.0604); Stage III, HR = 2.401 (95% CI: 1.441–4.000, *p* = 0.0008); Stage IV, HR = 5.241 (95% CI: 3.171–8.663, *p* < 0.0001). (B) Albumin (vs. Low): Medium, HR = 0.740 (95% CI: 0.615–0.891, *p* = 0.0015); High, HR = 0.439 (95% CI: 0.356–0.542, *p* < 0.0001). (C) Prealbumin (vs. Low): Medium, HR = 0.587 (95% CI: 0.486–0.709, *p* < 0.0001); High, HR = 0.449 (95% CI: 0.366–0.551, *p* < 0.0001). (D) HDL (vs. Low): Medium, HR = 0.631 (95% CI: 0.519–0.768, *p* < 0.0001); High, HR = 0.626 (95% CI: 0.515–0.761, *p* < 0.0001). (E) CRP (vs. Low): Medium, HR = 1.693 (95% CI: 1.362–2.105, *p* < 0.0001); High, HR = 2.374 (95% CI: 1.924–2.929, *p* < 0.0001). (F) Neutrophil count (vs. Low): Medium, HR = 1.469 (95% CI: 1.180–1.828, *p* = 0.0006); High, HR = 2.260 (95% CI: 1.838–2.779, *p* < 0.0001). The total number of events was 586 (34.2%). CRP, C‐reactive protein; HDL, high‐density lipoprotein; Neu, neutrophil; TNM, tumour, node, metastasis (staging system).

**Figure S11:** Screenshot of the interactive web‐based tool for cachexia risk and survival prediction.

## Data Availability

The data that support the findings of this study are available on request from the corresponding author. The data are not publicly available due to privacy or ethical restrictions.
