# Supplementary material for: Early Identification and Prognostic Stratification of Cancer Cachexia Using Explainable Machine Learning: A Multicentre Cohort Study
Source: J Cachexia Sarcopenia Muscle. 2026 Aug 21;17(4):e70370. doi: 10.1002/jcsm.70370 (PMC13498753; doi:10.1002/jcsm.70370)
Supplement: Supplementary file 1 [file JCSM-17-e70370-s005.docx]

**Supplementary Material**

**Supplementary Table 1. The baseline data of the population were followed up**

|  | **Follow-up** | **Cachexia** | **Non-cachexia** | **P value** |
| --- | --- | --- | --- | --- |
| N | 5172 | 1711 | 3461 |  |
| Sex |  |  |  |  |
| Man | 2970 (57.4%) | 1093 (63.9%) | 1877 (54.2%) | <0.001 |
| Women | 2202 (42.6%) | 618 (36.1%) | 1584 (45.8%) |  |
| Age | 59.1 [51.4, 65.5] | 59.2 [52.4, 66.1] | 59.1 [51.1, 65.2] | 0.017 |
| Smoking | 2397 (46.3%) | 858 (50.1%) | 1539 (44.5%) | <0.001 |
| Alcohol drinker | 1075 (20.8%) | 406 (23.7%) | 669 (19.3%) | <0.001 |
| Anticancer treatment |  |  |  |  |
| Surgery | 2482 (48.0%) | 794 (46.4%) | 1688 (48.8%) | 0.116 |
| Chemotherapy | 3276 (63.3%) | 999 (58.4%) | 2277 (65.8%) | <0.001 |
| Radiotherapy | 557 (10.8%) | 163 (9.5%) | 394 (11.4%) | 0.048 |
| Others | 3824 (73.9%) | 1181 (69%) | 2643 (76.4%) | <0.001 |
| Nutritional therapy |  |  |  |  |
| Parenteral | 332 (6.4%) | 181 (10.6%) | 151 (4.4%) | <0.001 |
| Enteral | 642 (12.4%) | 288 (16.8%) | 354 (10.2%) | <0.001 |
| None | 4239 (82%) | 1269 (74.2%) | 2970 (85.8%) | <0.001 |
| Tumor srage |  |  |  |  |
| 1 | 545 (10.5%) | 124 (7.2%) | 421 (12.2%) | <0.001 |
| 2 | 991 (19.2%) | 280 (16.4%) | 711 (20.5%) |  |
| 3 | 1813 (35.1%) | 650 (38%) | 1163 (33.6%) |  |
| 4 | 1823 (35.2%) | 657 (38.4%) | 1166 (33.7%) |  |
| PG-SGA score | 7.0 [3.0, 15.0] | 16.0 [11.0, 21.0] | 4.0 [1.0, 9.0] | <0.001 |
| Cachexia | 1711 (33.1%) | - | - |  |
| Survival time | 27.7 [14.8, 50.1] | 24.3 [12.9, 45.7] | 29.2 [16.1, 51.8] | <0.001 |
| CRP | 3.7 [2.8, 14.4] | 5.7 [3.0, 23.7] | 3.3 [2.4, 10.8] | <0.001 |
| RBC | 4.3 [3.8, 4.7] | 4.2 [3.7, 4.6] | 4.3 [3.9, 4.7] | <0.001 |
| WBC | 5.9 [4.6, 7.6] | 6.0 [4.7, 7.9] | 5.9 [4.6, 7.5] | 0.017 |
| PLT | 226.0 [176.0, 286.0] | 237.0 [184.0, 302.0] | 221.0 [174.0, 277.0] | <0.001 |
| Neutrophils | 3.5 [2.5, 5.0] | 3.6 [2.4, 5.3] | 3.5 [2.5, 4.8] | <0.001 |
| Lymphocyte | 1.5 [1.1, 1.9] | 1.5 [1.0, 1.8] | 1.6 [1.1, 2.0] | <0.001 |
| HB | 127.0 [113.0, 140.0] | 122.0 [107.0, 136.6] | 129.0 [116.0, 141.0] | <0.001 |
| ALT | 18.0 [12.4, 28.0] | 17.2 [11.4, 28.1] | 18.3 [13.0, 27.5] | 0.538 |
| AST | 21.2 [17.0, 28.0] | 21.0 [16.8, 29.0] | 21.5 [17.1, 27.4] | 0.025 |
| Total protein | 68.2 [63.8, 72.5] | 67.2 [62.5, 71.6] | 68.6 [64.5, 72.9] | <0.001 |
| Albumin | 39.1 [35.7, 42.0] | 37.8 [34.2, 41.0] | 39.6 [36.5, 42.3] | <0.001 |
| Prealbumin | 0.2 [0.2, 0.3] | 0.2 [0.1, 0.2] | 0.2 [0.2, 0.3] | 0.698 |
| Total bilirubin | 10.7 [8.0, 14.6] | 11.1 [8.1, 15.1] | 10.6 [7.9, 14.3] | <0.001 |
| Direct bilirubin | 2.9 [2.1, 4.0] | 3.1 [2.2, 4.4] | 2.8 [2.1, 3.9] | <0.001 |
| Total cholesterol | 4.5 [3.9, 5.3] | 4.4 [3.7, 5.1] | 4.6 [4.0, 5.3] | <0.001 |
| Triglyceride | 1.2 [0.9, 1.7] | 1.2 [0.9, 1.6] | 1.3 [1.0, 1.8] | <0.001 |
| HDL | 1.2 [1.0, 1.4] | 1.1 [0.9, 1.4] | 1.2 [1.0, 1.4] | <0.001 |
| LDL | 2.7 [2.2, 3.3] | 2.7 [2.1, 3.2] | 2.8 [2.2, 3.3] | 0.002 |
| Glucose | 5.3 [4.8, 6.0] | 5.2 [4.8, 5.9] | 5.3 [4.9, 6.0] | 0.146 |
| BUN | 5.1 [4.1, 6.3] | 5.0 [4.0, 6.3] | 5.2 [4.2, 6.3] | 0.802 |
| Creatinine | 66.0 [55.9, 79.0] | 67.3 [56.0, 81.4] | 65.5 [55.8, 78.0] | 0.002 |
| PLR | 149.0 [107.6, 215.6] | 165.6 [116.7, 237.3] | 140.8 [103.9, 204.9] | 0.001 |
| NLR | 2.3 [1.6, 3.6] | 2.5 [1.6, 4.1] | 2.2 [1.6, 3.3] | 0.001 |
| LLR | 3.8 [2.9, 5.4] | 4.1 [3.0, 6.2] | 3.7 [2.9, 5.1] | 0.101 |
| PNI | 46.9 [42.5, 50.6] | 45.3 [40.6, 49.4] | 47.6 [43.5, 51.2] | <0.001 |
| CAR | 0.1 [0.1, 0.4] | 0.2 [0.1, 0.7] | 0.1 [0.1, 0.3] | <0.001 |
| TyG | 8.6 [8.3, 9.0] | 8.5 [8.2, 8.9] | 8.6 [8.3, 9.0] | <0.001 |
| Height | 165.0 [159.0, 170.0] | 165.0 [160.0, 170.0] | 165.0 [158.0, 170.0] | 0.016 |
| Weight | 60.4 [54.0, 69.0] | 56.4 [50.0, 64.4] | 62.8 [56.0, 70.0] | <0.001 |
| BMI | 22.6 [20.3, 24.9] | 20.8 [18.9, 23.0] | 23.4 [21.2, 25.4] | <0.001 |
| MAC | 26.5 [24.0, 28.3] | 25.0 [23.0, 27.0] | 27.0 [25.0, 29.0] | <0.001 |
| TSF | 15.0 [10.0, 21.0] | 13.0 [9.0, 18.0] | 16.0 [11.0, 22.0] | <0.001 |
| HGS | 24.2 [18.3, 31.4] | 23.1 [17.1, 30.5] | 24.7 [19.0, 31.9] | <0.001 |
| MAMC | 21.5 [19.4, 23.4] | 20.9 [18.8, 22.8] | 21.7 [19.8, 23.7] | <0.001 |
| CAC | 33.5 [31.0, 36.0] | 32.3 [30.0, 34.5] | 34.0 [32.0, 37.0] | <0.001 |

ALT, alanine aminotransferase; AST, aspartate aminotransferase; BMI, body mass index; BUN, blood urea nitrogen; CAC, calf circumference; CAR, C-reactive protein-to-albumin ratio; CRP, C-reactive protein; HB, hemoglobin; HDL, high-density lipoprotein; HGS, handgrip strength; LDL, low-density lipoprotein; LLR, leukocyte-to-lymphocyte ratio; MAC, mid-arm circumference; MAMC, mid-arm muscle circumference; NLR, neutrophil-to-lymphocyte ratio; PG-SGA, patient-generated subjective global assessment; PLR, platelet-to-lymphocyte ratio; PLT, platelet count; PNI, prognostic nutritional index; RBC, red blood cell count; SII, systemic immune-inflammation index; TSF, triceps skinfold thickness; TyG, triglyceride–glucose index; WBC, white blood cell count.

Other anticancer treatments include endocrine therapy, targeted therapy, immunotherapy, interventional embolization, radiofrequency ablation, cryotherapy, and hyperthermia. Continuous variables are presented as median [interquartile range]. Categorical variables are expressed as frequency (percentage). Classification variables between groups using chi-square test. Continuous variables were compared between groups using t-test.

**Supplementary Table 2. Baseline characteristics of the Fujian external validation cohort**

|  | External validation set | high-prevalence group | low-prevalence group | P value |
| --- | --- | --- | --- | --- |
| N | 886 | 626 | 260 |  |
| Sex |  |  |  |  |
| Man | 595 (67.2%) | 433 (69.2%) | 162 (62.3%) | 0.057 |
| Women | 291 (32.8%) | 193 (30.8%) | 98 (37.7%) |  |
| Age | 58.3 [50.3, 66.3] | 58.5 [51.1, 66.4] | 57.9 [47.7, 66.2] | 0.222 |
| Smoking | 376 (42.4%) | 275 (43.9%) | 101 (38.8%) | 0.187 |
| Alcohol drinker | 162 (18.3%) | 133 (21.2%) | 29 (11.2%) | 0.001 |
| Anticancer treatment |  |  |  |  |
| Surgery | 454 (51.2%) | 357 (57%) | 97 (37.3%) | <0.001 |
| Chemotherapy | 360 (40.6%) | 274 (43.8%) | 86 (33.1%) | 0.004 |
| Radiotherapy | 65 (7.3%) | 38 (6.1%) | 27 (10.4%) | 0.036 |
| Others | 155 (17.5%) | 82 (13.1%) | 73 (28.1%) | <0.001 |
| Nutritional therapy |  |  |  |  |
| Parenteral | 81 (9.1%) | 71 (11.3%) | 10 (3.8%) | 0.001 |
| Enteral | 58 (6.5%) | 51 (8.1%) | 7 (2.7%) | 0.005 |
| None | 755 (85.2%) | 511 (81.6%) | 244 (93.8%) | <0.001 |
| Tumor srage |  |  |  |  |
| 1 | 40 (4.5%) | 23 (3.7%) | 17 (6.5%) | <0.001 |
| 2 | 115 (13%) | 84 (13.4%) | 31 (11.9%) |  |
| 3 | 278 (31.4%) | 229 (36.6%) | 49 (18.8%) |  |
| 4 | 453 (51.1%) | 290 (46.3%) | 163 (62.7%) |  |
| PG-SGA score | 7.0 [3.0, 14.0] | 9.0 [3.0, 15.0] | 4.0 [2.0, 11.0] | <0.001 |
| Cachexia | 316 (35.7%) | 262 (41.9%) | 54 (20.8%) | <0.001 |
| CRP | 3.6 [3.2, 18.1] | 3.5 [3.2, 15.0] | 4.6 [3.2, 24.1] | 0.011 |
| RBC | 4.2 [3.8, 4.6] | 4.2 [3.8, 4.5] | 4.3 [3.8, 4.6] | 0.316 |
| WBC | 6.1 [4.8, 8.0] | 5.9 [4.7, 7.6] | 6.7 [5.2, 8.4] | 0.006 |
| PLT | 237.0 [184.2, 292.8] | 234.5 [180.2, 290.8] | 242.5 [194.0, 298.5] | 0.210 |
| Neutrophils | 3.8 [2.8, 5.4] | 3.6 [2.7, 5.2] | 4.4 [3.2, 6.0] | 0.006 |
| Lymphocyte | 1.5 [1.1, 1.9] | 1.5 [1.1, 1.9] | 1.5 [1.1, 2.0] | 0.091 |
| HB | 123.0 [109.0, 136.0] | 122.0 [108.0, 134.0] | 126.0 [113.0, 140.0] | 0.002 |
| ALT | 20.0 [14.0, 30.0] | 20.0 [14.0, 29.8] | 20.0 [15.0, 31.2] | 0.036 |
| AST | 22.0 [17.0, 31.0] | 21.5 [17.0, 30.0] | 22.5 [17.0, 32.0] | 0.028 |
| Total protein | 69.3 [64.9, 73.8] | 69.0 [64.9, 73.6] | 69.9 [65.1, 74.9] | 0.239 |
| Albumin | 38.4 [34.8, 41.0] | 38.5 [34.7, 41.0] | 38.0 [35.1, 40.9] | 0.802 |
| Prealbumin | 0.2 [0.2, 0.3] | 0.2 [0.2, 0.3] | 0.2 [0.2, 0.3] | 0.484 |
| Total bilirubin | 10.8 [8.4, 14.6] | 11.0 [8.6, 14.9] | 10.4 [8.1, 14.1] | 0.884 |
| Direct bilirubin | 2.3 [1.7, 3.1] | 2.3 [1.7, 3.2] | 2.1 [1.5, 2.8] | 0.872 |
| Total cholesterol | 4.7 [4.1, 5.5] | 4.6 [4.0, 5.4] | 4.9 [4.2, 5.6] | 0.060 |
| Triglyceride | 1.2 [0.9, 1.7] | 1.1 [0.9, 1.5] | 1.3 [1.0, 1.9] | 0.001 |
| HDL | 1.1 [0.9, 1.4] | 1.1 [0.9, 1.4] | 1.2 [1.0, 1.4] | 0.449 |
| LDL | 3.1 [2.6, 3.8] | 3.1 [2.6, 3.7] | 3.2 [2.7, 3.8] | 0.156 |
| Glucose | 5.1 [4.7, 5.8] | 5.1 [4.7, 5.7] | 5.1 [4.7, 5.8] | 0.820 |
| BUN | 4.9 [3.8, 6.0] | 4.8 [3.7, 6.0] | 4.9 [4.0, 6.0] | 0.776 |
| Creatinine | 74.0 [64.0, 86.0] | 75.0 [65.0, 86.0] | 73.0 [62.0, 88.0] | 0.836 |
| PLR | 154.9 [115.0, 217.7] | 156.1 [116.7, 212.4] | 153.2 [112.1, 223.8] | 0.638 |
| NLR | 2.6 [1.8, 4.1] | 2.5 [1.7, 4.0] | 2.9 [2.0, 4.4] | 0.092 |
| LLR | 4.0 [3.1, 5.7] | 3.9 [3.0, 5.5] | 4.3 [3.3, 6.1] | 0.155 |
| PNI | 46.1 [41.7, 49.4] | 46.2 [41.4, 49.4] | 46.0 [42.4, 49.7] | 0.259 |
| CAR | 0.1 [0.1, 0.5] | 0.1 [0.1, 0.4] | 0.1 [0.1, 0.7] | 0.022 |
| TyG | 8.5 [8.2, 8.9] | 8.5 [8.2, 8.8] | 8.6 [8.3, 9.1] | <0.001 |
| Height | 165.0 [158.2, 170.0] | 165.0 [159.0, 170.0] | 163.0 [158.0, 170.0] | 0.049 |
| Weight | 59.0 [52.5, 66.5] | 59.0 [52.0, 66.0] | 59.0 [53.0, 67.0] | 0.078 |
| BMI | 21.9 [19.8, 24.1] | 21.8 [19.6, 23.9] | 22.2 [20.4, 24.8] | 0.001 |
| MAC | 26.0 [24.0, 28.0] | 26.0 [24.0, 28.0] | 26.5 [25.0, 28.5] | 0.002 |
| TSF | 12.0 [10.0, 19.0] | 12.0 [8.0, 18.0] | 14.0 [10.0, 20.0] | <0.001 |
| HGS | 23.9 [18.4, 31.2] | 24.0 [18.1, 31.2] | 23.9 [18.7, 31.7] | 0.751 |
| MAMC | 21.8 [19.7, 23.6] | 21.9 [19.7, 23.6] | 21.7 [19.7, 23.6] | 0.464 |
| CAC | 32.0 [30.0, 35.0] | 32.0 [30.0, 34.5] | 33.0 [31.0, 35.0] | 0.003 |
| AMA | 37.8 [30.9, 44.4] | 38.0 [30.9, 44.4] | 37.5 [31.0, 44.4] | 0.755 |

ALT, alanine aminotransferase; AST, aspartate aminotransferase; BMI, body mass index; BUN, blood urea nitrogen; CAC, calf circumference; CAR, C-reactive protein-to-albumin ratio; CRP, C-reactive protein; HB, hemoglobin; HDL, high-density lipoprotein; HGS, handgrip strength; LDL, low-density lipoprotein; LLR, leukocyte-to-lymphocyte ratio; MAC, mid-arm circumference; MAMC, mid-arm muscle circumference; NLR, neutrophil-to-lymphocyte ratio; PG-SGA, patient-generated subjective global assessment; PLR, platelet-to-lymphocyte ratio; PLT, platelet count; PNI, prognostic nutritional index; RBC, red blood cell count; SII, systemic immune-inflammation index; TSF, triceps skinfold thickness; TyG, triglyceride–glucose index; WBC, white blood cell count.

Other anticancer treatments include endocrine therapy, targeted therapy, immunotherapy, interventional embolization, radiofrequency ablation, cryotherapy, and hyperthermia. Continuous variables are presented as median [interquartile range]. Categorical variables are expressed as frequency (percentage). Classification variables between groups using chi-square test. Continuous variables were compared between groups using t-test.

**Supplementary Table 3. The distribution of different cancer types and the incidence rate of cachexia in this study**

| **Tumor type** | **Case (%)** | **Cachexia (%)** |
| --- | --- | --- |
| Pancreatic cancer | 246 (2.28) | 156 (63.41) |
| Cholangiocarcinoma | 162 (1.50) | 87 (53.70) |
| Gastric cancer | 1601 (14.83) | 846 (52.84) |
| Esophageal cancer | 475 (4.40) | 247 (52.00) |
| Colorectal cancer | 1869 (17.31) | 789 (42.22) |
| Gastric stromal tumor | 15 (0.14) | 6 (40.00) |
| Ovarian cancer | 272 (2.52) | 102 (37.50) |
| Malignant brain tumor | 24 (0.22) | 9 (37.50) |
| Cervix cancer | 176 (1.63) | 63 (35.80) |
| Liver cancer | 406 (3.76) | 145 (35.71) |
| Prostate cancer | 87 (0.81) | 30 (34.48) |
| Lung cancer | 3629 (33.61) | 1108 (30.53) |
| Nasopharyngeal carcinoma | 188 (1.74) | 57 (30.32) |
| Endometrial cancer | 83 (0.77) | 25 (30.12) |
| Bladder cancer | 86 (0.80) | 24 (27.91) |
| Breast cancer | 1141 (10.57) | 192 (16.83) |
| Other cancer | 434 (4.02) | 171 (39.40) |

**Supplementary Table 4. Baseline data for the high-prevalence and low-prevalence groups**

|  | **high-prevalence group** | **low-prevalence group** | **P value** |
| --- | --- | --- | --- |
| N | 4343 | 6453 |  |
| Sex |  |  | <0.001 |
| Man | 2906 (66.9%) | 3045 (47.2%) |  |
| Women | 1437 (33.1%) | 3408 (52.8%) |  |
| Age | 60.5 [53.2, 66.9] | 59.3 [52.1, 66.1] | <0.001 |
| Smoking | 1870 (43.1%) | 2889 (44.8%) | 0.082 |
| Alcohol drinker | 1037 (23.9%) | 1156 (17.9%) | <0.001 |
| Anticancer treatment |  |  |  |
| Surgery | 2634 (60.6%) | 2320 (36%) | <0.001 |
| Chemotherapy | 2760 (63.6%) | 4251 (65.9%) | 0.014 |
| Radiotherapy | 292 (6.7%) | 778 (12.1%) | <0.001 |
| Others | 2935 (67.6%) | 5427 (84.1%) | <0.001 |
| Nutritional therapy |  |  |  |
| Parenteral | 595 (13.7%) | 206 (3.2%) | <0.001 |
| Enteral | 1057 (24.3%) | 700 (10.8%) | <0.001 |
| None | 2845 (65.5%) | 5568 (86.3%) | <0.001 |
| Tumor srage |  |  | <0.001 |
| 1 | 289 (6.7%) | 772 (12%) |  |
| 2 | 804 (18.5%) | 1019 (15.8%) |  |
| 3 | 1795 (41.3%) | 1666 (25.8%) |  |
| 4 | 1455 (33.5%) | 2996 (46.4%) |  |
| PG-SGA score | 10.0 [3.0, 17.0] | 5.0 [2.0, 13.0] | <0.001 |
| Cachexia | 2114 (48.7%) | 1881 (29.1%) | <0.001 |
| CRP | 3.9 [2.0, 11.8] | 5.0 [2.3, 17.4] | <0.001 |
| RBC | 4.2 [3.8, 4.6] | 4.3 [3.8, 4.7] | <0.001 |
| WBC | 5.7 [4.5, 7.3] | 6.0 [4.8, 7.7] | <0.001 |
| PLT | 212.0 [165.0, 273.0] | 228.0 [177.0, 287.0] | <0.001 |
| Neutrophils | 3.3 [2.3, 4.8] | 3.8 [2.7, 5.2] | <0.001 |
| Lymphocyte | 1.4 [1.1, 1.9] | 1.5 [1.1, 1.9] | 0.003 |
| HB | 124.0 [109.0, 137.0] | 129.0 [116.0, 140.0] | <0.001 |
| ALT | 17.0 [11.8, 27.0] | 18.0 [12.5, 28.0] | 0.081 |
| AST | 21.0 [16.7, 29.0] | 21.3 [17.0, 28.0] | 0.134 |
| Total protein | 67.2 [62.5, 71.7] | 68.5 [64.0, 72.8] | <0.001 |
| Albumin | 39.0 [35.6, 42.3] | 39.1 [35.9, 42.1] | 0.707 |
| Prealbumin | 0.2 [0.2, 0.2] | 0.2 [0.2, 0.3] | 0.031 |
| Total bilirubin | 11.6 [8.8, 15.9] | 10.5 [8.0, 14.1] | <0.001 |
| Direct bilirubin | 3.1 [2.2, 4.6] | 2.6 [1.9, 3.7] | <0.001 |
| Total cholesterol | 4.4 [3.8, 5.2] | 4.7 [4.0, 5.4] | <0.001 |
| Triglyceride | 1.2 [0.9, 1.6] | 1.3 [1.0, 1.9] | <0.001 |
| HDL | 1.2 [1.0, 1.4] | 1.2 [1.0, 1.4] | <0.001 |
| LDL | 2.6 [2.1, 3.2] | 2.9 [2.3, 3.4] | <0.001 |
| Glucose | 5.3 [4.8, 6.0] | 5.4 [4.9, 6.1] | 0.002 |
| BUN | 5.1 [4.1, 6.2] | 5.2 [4.2, 6.3] | 0.2 |
| Creatinine | 67.3 [56.0, 80.0] | 63.2 [54.2, 74.8] | <0.001 |
| PLR | 148.8 [106.2, 216.3] | 153.3 [112.2, 214.7] | 0.088 |
| NLR | 2.3 [1.5, 3.7] | 2.5 [1.8, 3.8] | 0.066 |
| LLR | 3.8 [2.9, 5.6] | 4.0 [3.1, 5.5] | 0.026 |
| PNI | 46.5 [42.2, 50.5] | 46.8 [42.5, 50.6] | 0.055 |
| CAR | 0.1 [0.1, 0.3] | 0.1 [0.1, 0.5] | <0.001 |
| TyG | 8.6 [8.2, 8.9] | 8.7 [8.3, 9.1] | <0.001 |
| Height | 166.0 [160.0, 171.0] | 163.0 [158.0, 170.0] | <0.001 |
| Weight | 60.0 [52.5, 68.0] | 61.5 [55.0, 69.8] | <0.001 |
| BMI | 21.8 [19.7, 24.2] | 23.1 [20.8, 25.4] | <0.001 |
| MAC | 26.0 [24.0, 28.0] | 27.0 [25.0, 29.0] | <0.001 |
| TSF | 13.0 [9.0, 18.0] | 16.0 [12.0, 22.0] | <0.001 |
| HGS | 24.8 [18.3, 32.0] | 22.9 [17.6, 30.0] | <0.001 |
| MAMC | 21.6 [19.7, 23.7] | 21.7 [19.7, 23.7] | 0.416 |
| CAC | 34.0 [31.5, 36.5] | 34.1 [32.0, 37.0] | 0.174 |

ALT, alanine aminotransferase; AST, aspartate aminotransferase; BMI, body mass index; BUN, blood urea nitrogen; CAC, calf circumference; CAR, C-reactive protein-to-albumin ratio; CRP, C-reactive protein; HB, hemoglobin; HDL, high-density lipoprotein; HGS, handgrip strength; LDL, low-density lipoprotein; LLR, leukocyte-to-lymphocyte ratio; MAC, mid-arm circumference; MAMC, mid-arm muscle circumference; NLR, neutrophil-to-lymphocyte ratio; PG-SGA, patient-generated subjective global assessment; PLR, platelet-to-lymphocyte ratio; PLT, platelet count; PNI, prognostic nutritional index; RBC, red blood cell count; SII, systemic immune-inflammation index; TSF, triceps skinfold thickness; TyG, triglyceride–glucose index; WBC, white blood cell count.

Other anticancer treatments include endocrine therapy, targeted therapy, immunotherapy, interventional embolization, radiofrequency ablation, cryotherapy, and hyperthermia. Continuous variables are presented as median [interquartile range]. Categorical variables are expressed as frequency (percentage). Classification variables between groups using chi-square test. Continuous variables were compared between groups using t-test.

**Supplementary Table 5. Feature importance ranking for cachexia classification in the high-prevalence group.**

| **Variable** | **Single-factor LR** | **Multivariate LR** | **LASSO** | **XGBoost** | **RF** | **DT** | **Total** |
| --- | --- | --- | --- | --- | --- | --- | --- |
| BMI | 1 | 1 | 1 | 1 | 1 | 1 | 6 |
| HDL | 1 | 1 | 1 | 1 | 1 | 1 | 6 |
| CAC | 1 | 1 | 0 | 1 | 1 | 1 | 5 |
| EN | 1 | 1 | 1 | 0 | 1 | 1 | 5 |
| MAC | 1 | 0 | 0 | 0 | 1 | 1 | 3 |
| TyG | 1 | 1 | 1 | 0 | 0 | 0 | 3 |
| Albumin | 1 | 1 | 0 | 1 | 0 | 0 | 3 |
| PNI | 1 | 1 | 0 | 1 | 0 | 0 | 3 |
| HGS | 0 | 0 | 0 | 1 | 1 | 1 | 3 |
| HB | 1 | 1 | 0 | 0 | 0 | 1 | 3 |
| Other therapy | 0 | 0 | 1 | 0 | 1 | 0 | 2 |
| TSF | 0 | 0 | 0 | 1 | 0 | 1 | 2 |
| Surge | 0 | 0 | 1 | 0 | 1 | 0 | 2 |
| RBC | 1 | 0 | 0 | 0 | 0 | 1 | 2 |
| Radiotherapy | 0 | 0 | 1 | 0 | 0 | 0 | 1 |
| Total bilirubin | 0 | 0 | 0 | 1 | 0 | 0 | 1 |
| Alcohol drinker | 0 | 0 | 1 | 0 | 0 | 0 | 1 |
| PLT | 0 | 0 | 0 | 1 | 0 | 0 | 1 |
| CRP | 0 | 1 | 0 | 0 | 0 | 0 | 1 |
| Total cholesterol | 0 | 0 | 0 | 1 | 0 | 0 | 1 |
| Chemotherapy | 0 | 0 | 0 | 0 | 1 | 0 | 1 |
| Sex | 0 | 0 | 1 | 0 | 0 | 0 | 1 |
| MAMC | 0 | 0 | 0 | 0 | 0 | 1 | 1 |
| Smoking | 0 | 0 | 0 | 0 | 1 | 0 | 1 |

BMI, body mass index; CAC, calf circumference; CRP, C‑reactive protein; DT, decision tree; EN, enteral nutrition; HB, hemoglobin; HDL, high‑density lipoprotein (cholesterol); HGS, handgrip strength; LASSO, least absolute shrinkage and selection operator; LR, logistic regression; MAC, mid–upper arm circumference; MAMC, mid–upper arm muscle circumference; PLT, platelet count; PNI, prognostic nutritional index; RBC, red blood cell count; RF, random forest; TSF, triceps skinfold thickness; TyG, triglyceride–glucose index; XGBoost, eXtreme gradient boosting.

**Supplementary Table 6. Feature importance ranking for cachexia classification in the high-prevalence group.**

| **Variable** | **Single-factor LR** | **Multivariate LR** | **LASSO** | **XGBoost** | **RF** | **DT** | **Total** |
| --- | --- | --- | --- | --- | --- | --- | --- |
| BMI | 1 | 1 | 1 | 1 | 1 | 1 | 6 |
| CAC | 1 | 1 | 0 | 1 | 1 | 1 | 5 |
| CRP | 1 | 1 | 0 | 1 | 1 | 1 | 5 |
| MAC | 1 | 1 | 0 | 0 | 1 | 1 | 4 |
| CAR | 1 | 0 | 0 | 1 | 1 | 1 | 4 |
| Albumin | 1 | 1 | 0 | 1 | 1 | 0 | 4 |
| HDL | 0 | 0 | 1 | 1 | 0 | 1 | 3 |
| TSF | 1 | 1 | 0 | 0 | 1 | 0 | 3 |
| PNI | 1 | 1 | 0 | 0 | 1 | 0 | 3 |
| NLR | 1 | 0 | 0 | 0 | 1 | 1 | 3 |
| TNM | 0 | 1 | 1 | 0 | 0 | 0 | 2 |
| Neutrophils | 0 | 1 | 0 | 0 | 0 | 1 | 2 |
| Sex | 0 | 0 | 1 | 0 | 1 | 0 | 2 |
| PN | 0 | 0 | 1 | 0 | 0 | 0 | 1 |
| TyG | 0 | 0 | 1 | 0 | 0 | 0 | 1 |
| EN | 0 | 0 | 1 | 0 | 0 | 0 | 1 |
| Radiotherapy | 0 | 0 | 1 | 0 | 0 | 0 | 1 |
| WBC | 0 | 1 | 0 | 0 | 0 | 0 | 1 |
| Glucose | 0 | 0 | 0 | 1 | 0 | 0 | 1 |
| Triglyceride | 0 | 0 | 1 | 0 | 0 | 0 | 1 |
| Prealbumin | 0 | 0 | 0 | 0 | 0 | 1 | 1 |
| HGS | 0 | 0 | 0 | 1 | 0 | 0 | 1 |
| MAMC | 1 | 0 | 0 | 0 | 0 | 0 | 1 |
| RBC | 0 | 0 | 1 | 0 | 0 | 0 | 1 |
| BUN | 0 | 0 | 0 | 1 | 0 | 0 | 1 |
| LLR | 0 | 0 | 0 | 0 | 0 | 1 | 1 |
| PLR | 0 | 0 | 0 | 1 | 0 | 0 | 1 |

BMI, body mass index; BUN, blood urea nitrogen; CAC, calf circumference; CAR, C-reactive protein-to-albumin ratio; CRP, C-reactive protein; DT, decision tree; EN, enteral nutrition; HDL, high-density lipoprotein (cholesterol); HGS, handgrip strength; LASSO, least absolute shrinkage and selection operator; LLR, leukocyte-to-lymphocyte ratio; LR, logistic regression; MAC, mid–upper arm circumference; MAMC, mid–upper arm muscle circumference; NLR, neutrophil-to-lymphocyte ratio; PLR, platelet-to-lymphocyte ratio; PN, parenteral nutrition; PNI, prognostic nutritional index; RBC, red blood cell count; RF, random forest; TNM, Tumor–Node–Metastasis (staging system); TSF, triceps skinfold thickness; TyG, triglyceride–glucose index; WBC, white blood cell count; XGBoost, eXtreme gradient boosting.

**Supplementary Table 7. Feature importance ranking for the survival prediction model in cachexia patients.**

| **Variable** | **RSF** | **Lasso-Cox** | **XGBoost** | **Elastic.Net** | **GBM** | **Survival-SVM** | **Total** |
| --- | --- | --- | --- | --- | --- | --- | --- |
| TNM | 1 | 1 | 1 | 1 | 1 | 1 | 6 |
| CRP | 1 | 0 | 1 | 1 | 1 | 1 | 5 |
| Albumin | 1 | 1 | 0 | 1 | 0 | 1 | 4 |
| HDL | 1 | 1 | 0 | 1 | 0 | 1 | 4 |
| Prealbumin | 0 | 1 | 1 | 0 | 1 | 1 | 4 |
| ALT | 0 | 0 | 1 | 1 | 1 | 0 | 3 |
| AST | 0 | 1 | 0 | 1 | 0 | 1 | 3 |
| BMI | 1 | 0 | 1 | 0 | 1 | 0 | 3 |
| CAC | 1 | 1 | 0 | 0 | 0 | 1 | 3 |
| Neutrophils | 1 | 0 | 0 | 0 | 1 | 1 | 3 |
| RBC | 0 | 1 | 0 | 1 | 1 | 0 | 3 |
| Surgery | 0 | 1 | 0 | 1 | 0 | 1 | 3 |
| TSF | 1 | 0 | 0 | 1 | 1 | 0 | 3 |
| Age | 0 | 0 | 1 | 0 | 1 | 0 | 2 |
| Creatinine | 1 | 0 | 1 | 0 | 0 | 0 | 2 |
| Glucose | 0 | 0 | 1 | 0 | 1 | 0 | 2 |
| PLT | 0 | 1 | 1 | 0 | 0 | 0 | 2 |
| PNI | 0 | 0 | 0 | 1 | 0 | 1 | 2 |
| HB | 1 | 0 | 0 | 0 | 0 | 0 | 1 |
| Sex | 0 | 1 | 0 | 0 | 0 | 0 | 1 |
| Total cholesterol | 0 | 0 | 1 | 0 | 0 | 0 | 1 |

ALT, alanine aminotransferase; AST, aspartate aminotransferase; BMI, body mass index; CAC, calf circumference; CRP, C‑reactive protein; GBM, gradient boosting machine; HB, hemoglobin; HDL, high‑density lipoprotein (cholesterol); PLT, platelet count; PNI, prognostic nutritional index; RBC, red blood cell count; RSF, random survival forest; SVM, support vector machine; TNM, tumor–node–metastasis (staging system); TSF, triceps skinfold thickness; XGBoost, eXtreme gradient boosting.

**Supplementary Table 8. Variables selected for the prediction model in the High-Prevalence Group after feature screening.**

| **Variable** | **Multivariate.LR** | **LASSO** | **XGBoost** | **RF** | **DT** |
| --- | --- | --- | --- | --- | --- |
| BMI | 1 | 1 | 1 | 1 | 1 |
| CAC | 1 | 1 | 1 | 1 | 1 |
| EN | 1 | 1 | 1 | 1 | 1 |
| HB | 1 | 1 | 1 | 1 | 1 |
| HDL | 1 | 1 | 1 | 1 | 1 |
| HGS | 1 | 1 | 1 | 1 | 1 |
| MAC | 1 | 1 | 1 | 1 | 1 |
| TyG | 1 | 1 | 1 | 1 | 1 |
| PNI | 1 | 0 | 1 | 1 | 1 |
| Albumin | 0 | 1 | 0 | 0 | 0 |
| Radiotherapy | 0 | 1 | 0 | 0 | 0 |

BMI, body mass index; CAC, calf circumference; DT, decision tree; EN, enteral nutrition; HB, hemoglobin; HDL, high‑density lipoprotein; HGS, non‑dominant hand grip strength; LASSO, least absolute shrinkage and selection operator; LR, logistic regression; MAC, mid‑arm circumference; PNI, prognostic nutritional index; RF, random forest; TyG, triglyceride‑glucose index; XGBoost, eXtreme gradient boosting.

**Supplementary Table 9. Variables selected for the prediction model in the Low-Prevalence Group after feature screening.**

| **Variable** | **Multivariate.LR** | **LASSO** | **XGBoost** | **RF** | **DT** |
| --- | --- | --- | --- | --- | --- |
| BMI | 1 | 1 | 1 | 1 | 1 |
| CAC | 1 | 1 | 1 | 1 | 1 |
| CRP | 1 | 1 | 1 | 1 | 1 |
| HDL | 1 | 1 | 1 | 1 | 1 |
| MAC | 1 | 1 | 1 | 1 | 1 |
| TSF | 1 | 1 | 1 | 1 | 1 |
| NLR | 1 | 1 | 1 | 0 | 1 |
| Albumin | 1 | 0 | 1 | 0 | 1 |
| PN | 0 | 1 | 0 | 0 | 0 |
| PNI | 0 | 0 | 0 | 1 | 0 |
| TNM | 1 | 0 | 0 | 0 | 0 |
| TyG | 0 | 1 | 0 | 0 | 0 |

BMI, body mass index; CAC, cachexia stage; CRP, C‑reactive protein; DT, decision tree; HDL, high‑density lipoprotein; LASSO, least absolute shrinkage and selection operator; LR, logistic regression; MAC, mid‑arm circumference; NLR, neutrophil‑to‑lymphocyte ratio; PN, parenteral nutrition; PNI, prognostic nutritional index; RF, random forest; TNM, TNM staging; TSF, triceps skinfold thickness; TyG, triglyceride‑glucose index; XGBoost, eXtreme gradient boosting.

**Supplementary Table 10. Variables selected for the survival prediction model in cachexia patients.**

| **Variable** | **SRF** | **Lasso-Cox** | **XGBoost** | **Elastic.Net** | **GBM** | **Survival-SVM** |
| --- | --- | --- | --- | --- | --- | --- |
| Albumin | 1 | 1 | 1 | 1 | 1 | 1 |
| CRP | 1 | 1 | 1 | 1 | 1 | 1 |
| HDL | 1 | 1 | 1 | 1 | 1 | 1 |
| Prealbumin | 1 | 1 | 1 | 1 | 1 | 1 |
| TNM | 1 | 1 | 1 | 1 | 1 | 1 |
| ALT | 0 | 0 | 1 | 0 | 0 | 0 |
| Neutrophils | 1 | 0 | 0 | 0 | 0 | 0 |
| Surgery | 0 | 0 | 0 | 1 | 0 | 0 |
| TSF | 0 | 0 | 0 | 1 | 0 | 0 |

ALT, alanine aminotransferase; CRP, C-reactive protein; GBM, gradient boosting machine; HDL, high‑density lipoprotein; Lasso, least absolute shrinkage and selection operator; SRF, survival random forest; SVM, support vector machine; TNM, tumor, node, metastasis (staging system); TSF, triceps skinfold thickness; XGBoost, eXtreme gradient boosting.

**Supplementary Table 11.** **Predictive performance metrics of different machine learning algorithms under different variables**

| **Model** | **Multivariate LR** | **LASSO** | **XGBoost** | **RF** | **DT** |
| --- | --- | --- | --- | --- | --- |
| **High-prevalence group** | |  |  |  |  |
| **Variables set A** | |  |  |  |  |
| AUC | 0.704[0.689, 0.720] | 0.704[0.688, 0.719] | 0.803[0.790, 0.815] | 0.693[0.677, 0.708] | 0.665[0.651, 0.680] |
| Cutoff value | 0.483 | 0.480 | 0.484 | 0.479 | 0.457 |
| Sensitivity (Recall) | 0.676[0.605, 0.777] | 0.682[0.622, 0.773] | 0.766[0.721, 0.807] | 0.679[0.625, 0.723] | 0.621[0.600, 0.641] |
| Specificity | 0.638[0.532, 0.708] | 0.634[0.541, 0.697] | 0.694[0.651, 0.741] | 0.628[0.583, 0.681] | 0.665[0.645, 0.685] |
| Accuracy | 0.656[0.644, 0.671] | 0.657[0.644, 0.671] | 0.729[0.718, 0.743] | 0.653[0.640, 0.668] | 0.644[0.629, 0.657] |
| Precision | 0.639[0.606, 0.669] | 0.639[0.610, 0.664] | 0.704[0.680, 0.732] | 0.634[0.613, 0.661] | 0.638[0.616, 0.659] |
| F1 score | 0.657[0.629, 0.689] | 0.660[0.636, 0.687] | 0.733[0.716, 0.751] | 0.655[0.632, 0.675] | 0.629[0.611, 0.646] |
| **Variables set B** | |  |  |  |  |
| AUC | 0.705[0.689, 0.720] | 0.704[0.688, 0.719] | 0.867[0.857, 0.878] | 0.694[0.679, 0.710] | 0.665[0.651, 0.680] |
| Cutoff value | 0.470 | 0.477 | 0.541 | 0.463 | 0.457 |
| Sensitivity (Recall) | 0.695[0.563, 0.768] | 0.683[0.559, 0.768] | 0.706[0.680, 0.834] | 0.699[0.586, 0.763] | 0.621[0.600, 0.641] |
| Specificity | 0.615[0.541, 0.745] | 0.625[0.542, 0.750] | 0.846[0.721, 0.872] | 0.595[0.532, 0.710] | 0.665[0.645, 0.685] |
| Accuracy | 0.654[0.644, 0.670] | 0.653[0.642, 0.669] | 0.778[0.767, 0.791] | 0.646[0.636, 0.663] | 0.644[0.629, 0.657] |
| Precision | 0.631[0.610, 0.685] | 0.633[0.610, 0.678] | 0.813[0.734, 0.836] | 0.621[0.599, 0.664] | 0.638[0.616, 0.659] |
| F1 score | 0.662[0.611, 0.683] | 0.657[0.612, 0.684] | 0.756[0.744, 0.787] | 0.658[0.615, 0.682] | 0.629[0.611, 0.646] |
| **Low-prevalence group** | |  |  |  |  |
| **Variables set C** | |  |  |  |  |
| AUC | 0.735[0.721, 0.749] | 0.734[0.721, 0.748] | 0.839[0.829, 0.850] | 0.723[0.709, 0.737] | 0.633[0.621, 0.645] |
| Cutoff value | 0.336 | 0.304 | 0.296 | 0.355 | 0.413 |
| Sensitivity (Recall) | 0.591[0.535, 0.694] | 0.647[0.559, 0.677] | 0.753[0.725, 0.804] | 0.552[0.528, 0.690] | 0.366[0.345, 0.389] |
| Specificity | 0.760[0.658, 0.817] | 0.706[0.683, 0.792] | 0.758[0.709, 0.783] | 0.781[0.644, 0.803] | 0.900[0.892, 0.908] |
| Accuracy | 0.711[0.667, 0.737] | 0.689[0.677, 0.730] | 0.757[0.735, 0.772] | 0.714[0.656, 0.729] | 0.744[0.734, 0.755] |
| Precision | 0.503[0.450, 0.547] | 0.475[0.458, 0.536] | 0.561[0.526, 0.588] | 0.509[0.439, 0.536] | 0.601[0.574, 0.630] |
| F1 score | 0.544[0.528, 0.566] | 0.548[0.531, 0.567] | 0.643[0.628, 0.661] | 0.530[0.516, 0.553] | 0.455[0.434, 0.478] |
| **Variables set D** | |  |  |  |  |
| AUC | 0.735[0.722, 0.749] | 0.726[0.712, 0.740] | 0.832[0.821, 0.842] | 0.727[0.713, 0.741] | 0.633[0.621, 0.645] |
| Cutoff value | 0.347 | 0.333 | 0.297 | 0.301 | 0.413 |
| Sensitivity (Recall) | 0.573[0.555, 0.707] | 0.581[0.545, 0.665] | 0.754[0.728, 0.789] | 0.639[0.598, 0.662] | 0.366[0.345, 0.389] |
| Specificity | 0.784[0.654, 0.799] | 0.759[0.679, 0.794] | 0.749[0.715, 0.774] | 0.708[0.694, 0.746] | 0.900[0.892, 0.908] |
| Accuracy | 0.723[0.666, 0.734] | 0.707[0.673, 0.726] | 0.751[0.734, 0.765] | 0.688[0.677, 0.709] | 0.744[0.734, 0.755] |
| Precision | 0.522[0.451, 0.544] | 0.498[0.454, 0.532] | 0.553[0.527, 0.579] | 0.474[0.457, 0.504] | 0.601[0.574, 0.630] |
| F1 score | 0.547[0.533, 0.570] | 0.536[0.522, 0.559] | 0.638[0.623, 0.656] | 0.544[0.528, 0.564] | 0.455[0.434, 0.478] |
| **Variables set E** | |  |  |  |  |
| AUC | 0.736[0.722, 0.749] | 0.727[0.713, 0.741] | 0.806[0.794, 0.817] | 0.723[0.710, 0.737] | 0.633[0.621, 0.645] |
| Cutoff value | 0.339 | 0.313 | 0.381 | 0.325 | 0.413 |
| Sensitivity (Recall) | 0.585[0.557, 0.709] | 0.626[0.555, 0.669] | 0.665[0.628, 0.710] | 0.601[0.538, 0.665] | 0.366[0.345, 0.389] |
| Specificity | 0.771[0.646, 0.799] | 0.720[0.673, 0.786] | 0.797[0.759, 0.828] | 0.746[0.677, 0.804] | 0.900[0.892, 0.908] |
| Accuracy | 0.717[0.664, 0.734] | 0.692[0.673, 0.720] | 0.759[0.741, 0.773] | 0.704[0.674, 0.729] | 0.744[0.734, 0.755] |
| Precision | 0.512[0.451, 0.541] | 0.479[0.454, 0.520] | 0.574[0.540, 0.606] | 0.493[0.458, 0.534] | 0.601[0.574, 0.630] |
| F1 score | 0.546[0.533, 0.570] | 0.543[0.526, 0.562] | 0.616[0.601, 0.636] | 0.542[0.524, 0.561] | 0.455[0.434, 0.478] |
| **Variables set F** | |  |  |  |  |
| AUC | 0.735[0.722, 0.749] | 0.735[0.721, 0.749] | 0.809[0.798, 0.821] | 0.725[0.711, 0.739] | 0.633[0.621, 0.645] |
| Cutoff value | 0.336 | 0.319 | 0.315 | 0.333 | 0.413 |
| Sensitivity (Recall) | 0.592[0.554, 0.697] | 0.619[0.568, 0.690] | 0.763[0.645, 0.790] | 0.587[0.535, 0.664] | 0.366[0.345, 0.389] |
| Specificity | 0.763[0.659, 0.798] | 0.735[0.674, 0.785] | 0.695[0.671, 0.811] | 0.750[0.675, 0.799] | 0.900[0.892, 0.908] |
| Accuracy | 0.713[0.669, 0.731] | 0.702[0.673, 0.725] | 0.715[0.702, 0.764] | 0.702[0.669, 0.726] | 0.744[0.734, 0.755] |
| Precision | 0.506[0.452, 0.537] | 0.491[0.455, 0.528] | 0.507[0.488, 0.587] | 0.491[0.452, 0.529] | 0.601[0.574, 0.630] |
| F1 score | 0.546[0.531, 0.567] | 0.547[0.532, 0.569] | 0.609[0.595, 0.629] | 0.535[0.518, 0.555] | 0.455[0.434, 0.478] |
| **Variables set G** | |  |  |  |  |
| AUC | 0.737[0.724, 0.751] | 0.727[0.713, 0.741] | 0.870[0.861, 0.880] | 0.727[0.713, 0.741] | 0.633[0.621, 0.645] |
| Cutoff value | 0.340 | 0.313 | 0.279 | 0.325 | 0.413 |
| Sensitivity (Recall) | 0.583[0.559, 0.748] | 0.626[0.555, 0.669] | 0.817[0.776, 0.838] | 0.602[0.533, 0.671] | 0.366[0.345, 0.389] |
| Specificity | 0.775[0.607, 0.797] | 0.720[0.673, 0.786] | 0.756[0.741, 0.795] | 0.744[0.678, 0.812] | 0.900[0.892, 0.908] |
| Accuracy | 0.719[0.649, 0.732] | 0.692[0.673, 0.720] | 0.774[0.764, 0.794] | 0.703[0.674, 0.734] | 0.744[0.734, 0.755] |
| Precision | 0.516[0.439, 0.540] | 0.479[0.454, 0.520] | 0.579[0.561, 0.616] | 0.492[0.456, 0.542] | 0.601[0.574, 0.630] |
| F1 score | 0.547[0.534, 0.570] | 0.543[0.526, 0.562] | 0.678[0.664, 0.697] | 0.542[0.526, 0.562] | 0.455[0.434, 0.478] |

The analysis employed seven distinct variable sets (A–G). Each set consisted of a core group of variables plus the top three most important features identified by each individual machine-learning model in the preliminary training round.

Set A: BMI, HDL, CAC, EN, MAC, TyG, HGS, HB, Albumin.

Set B: BMI, HDL, CAC, EN, MAC, TyG, HGS, HB, PNI.

Set C: BMI, CAC, MAC, HDL, TSF, CAR, NLR.

Set D: BMI, CAC, MAC, HDL, TSF, CRP, PNI.

Set E: BMI, CAC, MAC, HDL, TSF, CRP, Albumin.

Set F: BMI, CAC, MAC, HDL, TSF, CRP, NLR.

Set G: BMI, CAC, MAC, HDL, TSF, CRP, Albumin, NLR.

AUC, area under the (receiver operating characteristic) curve; BMI, body mass index; CAC, cachexia stage; CAR, C‑reactive protein‑to‑albumin ratio; CRP, C‑reactive protein; DT, decision tree; EN, enteral nutrition; HB, hemoglobin; HDL, high‑density lipoprotein; HGS, hand grip strength; LASSO, least absolute shrinkage and selection operator; LR, logistic regression; MAC, mid‑arm circumference; NLR, neutrophil‑to‑lymphocyte ratio; PNI, prognostic nutritional index; RF, random forest; TSF, triceps skinfold thickness; TyG, triglyceride‑glucose index; XGBoost, eXtreme gradient boosting.

**Supplementary Table 12. Subgroup analysis of the XGBoost-based cachexia classification model in the High- and Low-Prevalence Groups.**

| **Subgroup** | **Level** | **n** | **n (cachexia)** | **n (non-cachexia)** | **AUC** | **Cutoff value** | **Sensitivity**  **(Recall)** | **Specificity** | **Accuracy** | **Precision** | **F1 score** |
| --- | --- | --- | --- | --- | --- | --- | --- | --- | --- | --- | --- |
| **High-prevalence group** | | |  |  |  |  |  |  |  |  |  |
| Age (year) | <60 | 2072 | 1000 | 1072 | 0.862[0.847, 0.877] | 0.470 | 0.796[0.676, 0.859] | 0.746[0.685, 0.868] | 0.770[0.757, 0.791] | 0.745[0.710, 0.831] | 0.770[0.736, 0.794] |
|  | >=60 | 2271 | 1114 | 1157 | 0.872[0.858, 0.886] | 0.533 | 0.733[0.661, 0.792] | 0.836[0.778, 0.907] | 0.786[0.772, 0.805] | 0.811[0.774, 0.877] | 0.770[0.745, 0.793] |
| Sex | Male | 2906 | 1445 | 1461 | 0.864[0.851, 0.877] | 0.527 | 0.717[0.667, 0.835] | 0.833[0.719, 0.878] | 0.775[0.763, 0.792] | 0.809[0.741, 0.848] | 0.760[0.742, 0.794] |
|  | Female | 1437 | 669 | 768 | 0.875[0.858, 0.892] | 0.540 | 0.726[0.653, 0.886] | 0.835[0.675, 0.902] | 0.784[0.763, 0.808] | 0.793[0.693, 0.858] | 0.758[0.733, 0.797] |
| Surgery | No | 1709 | 807 | 902 | 0.861[0.844, 0.878] | 0.540 | 0.703[0.627, 0.825] | 0.841[0.721, 0.905] | 0.776[0.757, 0.796] | 0.799[0.718, 0.860] | 0.748[0.720, 0.778] |
|  | Yes | 2634 | 1307 | 1327 | 0.872[0.859, 0.885] | 0.532 | 0.721[0.688, 0.867] | 0.840[0.695, 0.876] | 0.781[0.771, 0.798] | 0.816[0.732, 0.849] | 0.766[0.754, 0.805] |
| Radiotherapy | No | 4051 | 1998 | 2053 | 0.866[0.855, 0.877] | 0.531 | 0.720[0.669, 0.836] | 0.828[0.713, 0.878] | 0.775[0.763, 0.789] | 0.803[0.732, 0.844] | 0.759[0.742, 0.788] |
|  | Yes | 292 | 116 | 176 | 0.884[0.845, 0.922] | 0.543 | 0.733[0.654, 0.885] | 0.881[0.736, 0.941] | 0.822[0.774, 0.870] | 0.802[0.667, 0.897] | 0.766[0.713, 0.830] |
| Chemotherapy | No | 1583 | 696 | 887 | 0.892[0.876, 0.907] | 0.457 | 0.845[0.678, 0.881] | 0.762[0.727, 0.921] | 0.798[0.783, 0.828] | 0.736[0.706, 0.876] | 0.787[0.756, 0.810] |
|  | Yes | 2760 | 1418 | 1342 | 0.851[0.837, 0.865] | 0.542 | 0.697[0.674, 0.807] | 0.834[0.722, 0.855] | 0.763[0.750, 0.781] | 0.816[0.753, 0.838] | 0.752[0.736, 0.781] |
| BMI | <18.5 | 590 | 414 | 176 | 0.926[0.905, 0.948] | 0.663 | 0.831[0.779, 0.882] | 0.886[0.822, 0.941] | 0.847[0.813, 0.880] | 0.945[0.920, 0.971] | 0.884[0.856, 0.910] |
|  | >24 | 1130 | 364 | 766 | 0.872[0.851, 0.893] | 0.339 | 0.819[0.714, 0.870] | 0.762[0.719, 0.868] | 0.781[0.757, 0.816] | 0.621[0.576, 0.719] | 0.706[0.674, 0.743] |
|  | 18.5-24 | 2623 | 1336 | 1287 | 0.859[0.845, 0.873] | 0.541 | 0.722[0.668, 0.766] | 0.828[0.788, 0.880] | 0.774[0.761, 0.791] | 0.813[0.782, 0.856] | 0.764[0.743, 0.788] |
| Nutritional therapy | PN+EN | 194 | 108 | 86 | 0.939[0.908, 0.970] | 0.624 | 0.870[0.776, 0.957] | 0.872[0.774, 0.963] | 0.871[0.830, 0.918] | 0.895[0.826, 0.966] | 0.883[0.838, 0.928] |
|  | EN | 863 | 541 | 322 | 0.895[0.874, 0.915] | 0.590 | 0.795[0.713, 0.887] | 0.817[0.722, 0.896] | 0.803[0.772, 0.840] | 0.879[0.837, 0.925] | 0.835[0.794, 0.873] |
|  | PN | 401 | 174 | 227 | 0.927[0.904, 0.951] | 0.434 | 0.856[0.805, 0.950] | 0.837[0.735, 0.895] | 0.845[0.810, 0.880] | 0.801[0.714, 0.868] | 0.828[0.792, 0.870] |
|  | Neither | 2885 | 1291 | 1594 | 0.844[0.830, 0.858] | 0.464 | 0.746[0.645, 0.865] | 0.760[0.639, 0.862] | 0.754[0.734, 0.772] | 0.716[0.656, 0.792] | 0.731[0.710, 0.759] |
| **Low-prevalence group** | |  |  |  |  |  |  |  |  |  |  |
| Age (year) | <60 | 3389 | 934 | 2455 | 0.873[0.860, 0.885] | 0.292 | 0.763[0.734, 0.862] | 0.813[0.715, 0.841] | 0.800[0.752, 0.819] | 0.609[0.529, 0.646] | 0.677[0.650, 0.701] |
|  | >=60 | 3064 | 947 | 2117 | 0.869[0.855, 0.882] | 0.282 | 0.847[0.772, 0.872] | 0.718[0.694, 0.791] | 0.758[0.743, 0.787] | 0.573[0.550, 0.626] | 0.684[0.664, 0.708] |
| Sex | Male | 3045 | 976 | 2069 | 0.872[0.859, 0.885] | 0.292 | 0.833[0.750, 0.877] | 0.736[0.694, 0.820] | 0.767[0.745, 0.803] | 0.598[0.562, 0.670] | 0.696[0.676, 0.722] |
|  | Female | 3408 | 905 | 2503 | 0.868[0.855, 0.881] | 0.280 | 0.775[0.731, 0.832] | 0.796[0.736, 0.838] | 0.790[0.758, 0.814] | 0.578[0.527, 0.629] | 0.662[0.638, 0.690] |
| Surgery | No | 4133 | 1311 | 2822 | 0.867[0.856, 0.879] | 0.292 | 0.834[0.741, 0.872] | 0.728[0.697, 0.819] | 0.762[0.746, 0.799] | 0.588[0.561, 0.663] | 0.690[0.674, 0.711] |
|  | Yes | 2320 | 570 | 1750 | 0.876[0.860, 0.891] | 0.248 | 0.798[0.706, 0.867] | 0.784[0.708, 0.873] | 0.787[0.747, 0.836] | 0.546[0.484, 0.650] | 0.649[0.617, 0.686] |
| Radiotherapy | No | 5675 | 1668 | 4007 | 0.866[0.856, 0.876] | 0.281 | 0.805[0.766, 0.831] | 0.757[0.734, 0.794] | 0.771[0.759, 0.791] | 0.580[0.558, 0.616] | 0.674[0.660, 0.694] |
|  | Yes | 778 | 213 | 565 | 0.903[0.881, 0.925] | 0.294 | 0.869[0.824, 0.937] | 0.789[0.727, 0.836] | 0.811[0.775, 0.844] | 0.609[0.545, 0.677] | 0.716[0.677, 0.765] |
| Chemotherapy | No | 2202 | 717 | 1485 | 0.877[0.862, 0.892] | 0.325 | 0.787[0.761, 0.865] | 0.805[0.725, 0.826] | 0.799[0.766, 0.817] | 0.661[0.595, 0.692] | 0.718[0.692, 0.745] |
|  | Yes | 4251 | 1164 | 3087 | 0.866[0.854, 0.877] | 0.279 | 0.796[0.761, 0.850] | 0.767[0.709, 0.804] | 0.775[0.746, 0.797] | 0.564[0.519, 0.605] | 0.660[0.639, 0.684] |
| BMI | <18.5 | 487 | 320 | 167 | 0.911[0.885, 0.937] | 0.580 | 0.897[0.776, 0.934] | 0.772[0.729, 0.905] | 0.854[0.805, 0.887] | 0.883[0.858, 0.944] | 0.890[0.840, 0.916] |
|  | >24 | 2507 | 401 | 2106 | 0.886[0.870, 0.902] | 0.177 | 0.838[0.796, 0.911] | 0.758[0.678, 0.802] | 0.771[0.715, 0.800] | 0.397[0.340, 0.440] | 0.539[0.493, 0.578] |
|  | 18.5-24 | 3459 | 1160 | 2299 | 0.847[0.833, 0.860] | 0.349 | 0.732[0.650, 0.850] | 0.784[0.663, 0.867] | 0.766[0.724, 0.800] | 0.631[0.557, 0.716] | 0.678[0.662, 0.700] |
| Nutritional therapy | PN+EN | 73 | 40 | 33 | 0.871[0.790, 0.953] | 0.453 | 0.775[0.568, 0.949] | 0.848[0.703, 1.000] | 0.808[0.740, 0.918] | 0.861[0.763, 1.000] | 0.816[0.716, 0.919] |
|  | EN | 627 | 236 | 391 | 0.882[0.856, 0.909] | 0.320 | 0.788[0.683, 0.881] | 0.808[0.723, 0.909] | 0.801[0.767, 0.842] | 0.713[0.641, 0.828] | 0.748[0.712, 0.795] |
|  | PN | 133 | 77 | 56 | 0.938[0.899, 0.977] | 0.363 | 0.896[0.779, 0.960] | 0.875[0.797, 0.967] | 0.887[0.835, 0.940] | 0.908[0.849, 0.974] | 0.902[0.846, 0.947] |
|  | Neither | 5620 | 1528 | 4092 | 0.864[0.854, 0.874] | 0.282 | 0.802[0.774, 0.850] | 0.763[0.714, 0.789] | 0.774[0.751, 0.789] | 0.559[0.525, 0.586] | 0.659[0.640, 0.677] |

AUC, area under the (receiver operating characteristic) curve; BMI, body mass index; EN, enteral nutrition; PN, parenteral nutrition.

**Supplementary Figures**

**Supplementary Fig 1.** Flowchart of participant selection and data preprocessing from the INSCOC database. INSCOC, Investigation on Nutrition Status and Clinical Outcome of Common Cancers.

**Supplementary Fig 2.** SHAP dependence plots for key features in the XGBoost-based cachexia classification model (high-prevalence group). (A) Dependence of SHAP values on BMI. (B) Dependence of SHAP values on HDL. (C) Dependence of SHAP values on EN. (D) Dependence of SHAP values on CAC. (E) Dependence of SHAP values on MAC. BMI, body mass index; CAC, calf circumference; EN, enteral nutrition; HDL, high-density lipoprotein; MAC, mid-arm circumference; SHAP, SHapley additive explanations; XGBoost, eXtreme gradient boosting.

**Supplementary Fig 3.** SHAP dependence plots for key features in the XGBoost-based cachexia classification model (high-prevalence group). (A) Dependence of SHAP values on PNI. (B) Dependence of SHAP values on HDL. (C) Dependence of SHAP values on TyG. (D) Dependence of SHAP values on HB. (E) Dependence of SHAP values on HGS. HB, hemoglobin; HGS, hand grip strength; PNI, prognostic nutritional index; SHAP, SHapley additive explanations; TyG, triglyceride-glucose index; XGBoost, eXtreme gradient boosting.

**Supplementary Fig 4.** SHAP dependence plots for key features in the XGBoost-based cachexia classification model (low-prevalence group). (A) Dependence of SHAP values on NLR. (B) Dependence of SHAP values on BMI. (C) Dependence of SHAP values on TSF. (D) Dependence of SHAP values on CRP. BMI, body mass index; CRP, C-reactive protein; NLR, neutrophil-to-lymphocyte ratio; SHAP, SHapley additive explanations; TSF, triceps skinfold thickness; XGBoost, eXtreme gradient boosting.

**Supplementary Fig 5.** SHAP dependence plots for key features in the XGBoost-based cachexia classification model (low-prevalence group). (A) Dependence of SHAP values on Albumin. (B) Dependence of SHAP values on HDL. (C) Dependence of SHAP values on MAC. (D) Dependence of SHAP values on CAC. CAC, calf circumference; HDL, high-density lipoprotein; MAC, mid-arm circumference; SHAP, SHapley additive explanations; XGBoost, eXtreme gradient boosting.

**Supplementary Fig 6.** Case-level interpretation of the XGBoost classifier using SHAP force plots in the high- and low-prevalence groups. (A) A cachexia case from the high-prevalence group. (B) A cachexia case from the low-prevalence group. (C) A non-cachexia case from the high-prevalence group. (D) A non-cachexia case from the low-prevalence group. BMI, body mass index; CAC, calf circumference; CRP, C-reactive protein; EN, enteral nutrition; HB, hemoglobin; HDL, high-density lipoprotein; HGS, hand grip strength; MAC, mid-arm circumference; NLR, neutrophil-to-lymphocyte ratio; PNI, prognostic nutritional index; SHAP, SHapley additive explanations; TSF, triceps skinfold thickness; TyG, triglyceride-glucose index.

**Supplementary Fig 7.** Calibration and clinical utility of the RSF survival prediction model across different time points. (A) Calibration plot at 3 months. (B) Decision curve analysis at 3 months. (C) Calibration plot at 6 months. (D) Decision curve analysis at 6 months. (E) Calibration plot at 9 months. (F) Decision curve analysis at 9 months. RSF, random survival forest.

**Supplementary Fig 8.** Calibration and clinical utility of the GBM survival prediction model across different time points. (A) Calibration plot at 3 months. (B) Decision curve analysis at 3 months. (C) Calibration plot at 6 months. (D) Decision curve analysis at 6 months. (E) Calibration plot at 9 months. (F) Decision curve analysis at 9 months. RSF, Random Survival Forest. GBM, gradient boosting machine

**Supplementary Fig 9.** Interpretability of the survival prediction model: feature importance and partial dependence plots. (A) Variable importance ranking of the Random Survival Forest (RSF)-based model. (B–G) Partial dependence plots showing the marginal effect on the predicted 12‑month survival probability for: (B) TNM stage, (C) albumin, (D) prealbumin, (E) HDL, (F) CRP, and (G) neutrophil count. CRP, C‑reactive protein; HDL, high‑density lipoprotein; Neu, neutrophil; RSF, random survival forest; TNM, Tumor, Node, Metastasis (staging system); VIMP, variable importance.

**Supplementary Fig 10.** Prognostic stratification using Kaplan–Meier survival analysis according to clinical variables. (A) Stratified by TNM stage. (B–F) Stratified by tertiles (low, medium, high) of: (B) albumin, (C) prealbumin, (D) HDL, (E) CRP, and (F) neutrophil count. The hazard ratios (HRs), 95% confidence intervals (CIs), and p-values were derived from univariate Cox regression analyses with the reference groups as indicated. (A) TNM stage (vs. Stage I): Stage II, HR=1.695 (95% CI: 0.977–2.939, p=0.0604); Stage III, HR=2.401 (95% CI: 1.441–4.000, p=0.0008); Stage IV, HR=5.241 (95% CI: 3.171–8.663, p<0.0001). (B) Albumin (vs. Low): Medium, HR=0.740 (95% CI: 0.615–0.891, p=0.0015); High, HR=0.439 (95% CI: 0.356–0.542, p<0.0001). (C) Prealbumin (vs. Low): Medium, HR=0.587 (95% CI: 0.486–0.709, p<0.0001); High, HR=0.449 (95% CI: 0.366–0.551, p<0.0001). (D) HDL (vs. Low): Medium, HR=0.631 (95% CI: 0.519–0.768, p<0.0001); High, HR=0.626 (95% CI: 0.515–0.761, p<0.0001). (E) CRP (vs. Low): Medium, HR=1.693 (95% CI: 1.362–2.105, p<0.0001); High, HR=2.374 (95% CI: 1.924–2.929, p<0.0001). (F) Neutrophil count (vs. Low): Medium, HR=1.469 (95% CI: 1.180–1.828, p=0.0006); High, HR=2.260 (95% CI: 1.838–2.779, p<0.0001). The total number of events was 586 (34.2%). CRP, C‑reactive protein; HDL, high‑density lipoprotein; Neu, neutrophil; TNM, Tumor, Node, Metastasis (staging system).

**Supplementary Fig 11. Screenshot of the interactive web‑based tool for cachexia risk and survival prediction.**
